# Competing subclones and fitness diversity shape tumor evolution across cancer types

**DOI:** 10.1093/bioinformatics/btag127

**Published:** 2026-03-13

**Authors:** Hai Chen, Jingmin Shu, Rekha Mudappathi, Elaine Li, Panwen Wang, Leif Bergsagel, Ping Yang, Zhifu Sun, Logan Zhao, Changxin Shi, Jeffrey P Townsend, Carlo Maley, Li Liu

**Affiliations:** College of Health Solutions, Arizona State University, Phoenix, AZ 85004, United States; Biodesign Institute, Arizona State University, Tempe, AZ 85281, United States; Division of Epidemiology, Department of Quantitative Health Sciences, Mayo Clinic, Scottsdale, AZ 85259, United States; College of Health Solutions, Arizona State University, Phoenix, AZ 85004, United States; Biodesign Institute, Arizona State University, Tempe, AZ 85281, United States; College of Health Solutions, Arizona State University, Phoenix, AZ 85004, United States; Biodesign Institute, Arizona State University, Tempe, AZ 85281, United States; Division of Epidemiology, Department of Quantitative Health Sciences, Mayo Clinic, Scottsdale, AZ 85259, United States; Emory University, Atlanta, GA 30322, United States; Department of Quantitative Health Sciences, Mayo Clinic, Scottsdale, AZ 85259, United States; Comprehensive Cancer Center, Mayo Clinic, Scottsdale, AZ 85054, United States; Division of Epidemiology, Department of Quantitative Health Sciences, Mayo Clinic, Scottsdale, AZ 85259, United States; Division of Computational Biology, Department of Quantitative Health Sciences, Mayo Clinic, Rochester, MN 55905, United States; Division of Hematology/Oncology, Department of Medicine, Mayo Clinic, Scottsdale, AZ 85259, United States; Division of Hematology/Oncology, Department of Medicine, Mayo Clinic, Scottsdale, AZ 85259, United States; Department of Biostatistics, Yale School of Public Health, New Haven, CT 06520, United States; Department of Ecology and Evolutionary Biology, Yale University, New Haven, CT 06520, United States; Program in Computational Biology and Biomedical Informatics, Yale University, New Haven, CT 06520, United States; Yale Cancer Center, Yale University, New Haven, CT 06519, United States; Biodesign Institute, Arizona State University, Tempe, AZ 85281, United States; Arizona Cancer Evolution Center, Arizona State University, Tempe, AZ 85281, United States; College of Health Solutions, Arizona State University, Phoenix, AZ 85004, United States; Biodesign Institute, Arizona State University, Tempe, AZ 85281, United States

## Abstract

**Motivation:**

Intratumor heterogeneity arises from ongoing somatic evolution and complicates cancer diagnosis, prognosis, and treatment. Reconstructing evolutionary dynamics typically requires spatiotemporal samples, which are often unavailable in clinical settings. Computational approaches that can infer tumor evolutionary history from single-timepoint bulk sequencing data remain limited.

**Results:**

We present es*t*imating *e*volution*a*ry events *t*hrough s*i*ngle-ti*m*epoint s*e*quencing (TEATIME), a novel computational framework that models tumors as mixtures of two competing cell populations: an ancestral clone with baseline fitness and a derived subclone with elevated fitness. Using cross-sectional bulk sequencing data, TEATIME estimates mutation rates, timing of subclone emergence, relative fitness, and number of generations of growth. To quantify intratumor fitness asymmetries, we introduce a novel metric—fitness diversity—which captures the imbalance between competing cell populations and serves as a measure of functional intratumor heterogeneity. Applying TEATIME to 33 tumor types from The Cancer Genome Atlas, we revealed divergent as well as convergent evolutionary patterns. Notably, we found that immune-hot microenvironments constraint subclonal expansion and limit fitness diversity. Moreover, we detected temporal dependencies in mutation acquisition, where early driver mutations in ancestral clones epistatically shape the fitness landscape, predisposing specific subclones to selective advantages. These findings underscore the importance of intratumor competition and tumor-microenvironment interactions in shaping evolutionary trajectories, driving intratumor heterogeneity. Lastly, we demonstrate that TEATIME-derived evolutionary parameters and fitness diversity offer novel prognostic insights across multiple cancer types.

**Availability and implementation:**

R implementation of TEATIME is available on GitHub (https://github.com/liliulab/TEATIME) and Zenodo (https://zenodo.org/records/17422174).

## 1 Introduction

A tumor is composed of diverse cell populations, each with distinct molecular and phenotypic profiles (Gay *et al.* 2016, Prasetyanti and Medema 2017). This diversity enables a tumor to adapt to various selection pressures, such as changes in the microenvironment and treatment, leading to proliferation of subpopulations with greater fitness (Schmitt *et al.* 2016, Visser and Joyce 2023). Understanding the evolutionary parameters governing proliferation and survival of these subpopulations is crucial for gaining insights into tumor development, metastasis, therapy resistance, and disease recurrence.

The clonal evolution model posits that a tumor originates from a single cell that has acquired genetic or epigenetic alterations conferring a growth advantage. As this cell proliferates, it forms a population (i.e. clone) that can further evolve by accumulating new alterations. While most of these alterations are selectively neutral, some provide additional growth advantages (Cannataro *et al.* 2018), leading to the emergence of subpopulations (i.e. subclones) with increased fitness (Qiao *et al.* 2014, Tarabichi *et al.* 2021). This evolutionary process continuously shapes the cellular composition of a tumor, resulting in complex intratumor heterogeneity.

Tumor evolutionary processes are often reconstructed by analyzing somatically acquired single nucleotide alterations (SNAs), owing to their high abundance, close adherence to the infinite-site mutation model, and relatively stable mutation rates. Although SNAs do not account for all tumorigenic events, they constitute many key driver alterations and may serve as a reliable proxy. Qualitative analyses of tumor evolution typically categorize SNAs into early versus late, or clonal versus subclonal groups (Alexandrov *et al.* 2020, Gerstung *et al.* 2020, Fontana *et al.* 2023). This temporal order has been associated with molecular and phenotypic traits of tumors. Quantitative approaches estimate key evolutionary parameters such as mutation rates and relative fitness of subclones, and cellular composition metrics, offering a more dynamic and mechanistic understanding of tumor development. Williams *et al.* pioneered a mathematical model of clonal evolution to estimate evolutionary parameters using variant allele frequencies (VAFs) obtained from whole-exome sequencing (WES) or whole-genome sequencing (WGS) data (Williams *et al.* 2016). Building on this foundation, several subsequent studies have explored machine learning and other techniques to enhance the estimations (Williams *et al.* 2018, Caravagna *et al.* 2020, Ouellette and Awadalla 2022). However, these analyses have relied on one or both of the following assumptions: first, the tumor cell population size at the time of sampling is known; and second, the number and VAF of low-frequency variants follow a power-law distribution (Williams *et al.* 2016), although these variants are polyphyletic and do not share a common trajectory. These simplifications can lead to model convergence failures in many tumors, compromise the accuracy of the estimates, and limit the biological relevance of the results.

To address these limitations, we developed a method named es*t*imating *e*volution*a*ry events *t*hrough s*i*ngle-ti*m*epoint s*e*quencing (TEATIME). This method models the tumor cell population size as a dynamic variable, tracks the temporal changes of VAFs as the tumor evolves, and jointly infers mutation clusters and evolutionary parameters in an iterative framework. Extensive simulations demonstrated that TEATIME enhanced the accuracy and robustness of parameter estimation generally. When applied to WES data from 33 diverse tumor types cataloged in The Cancer Genome Atlas (TCGA), TEATIME increased the number of analyzable tumors by 2- to 37-fold compared to existing methods. TEATIME also revealed striking divergences and conserved patterns in evolutionary profiles. Furthermore, we introduce a novel measure, fitness diversity, that captures the imbalance between subclones with distinct fitness levels. Fitness diversity represents a new conceptual dimension regarding intratumor heterogeneity. By linking quantitative evolutionary parameters with immune context, pathway activity, and patient prognosis, TEATIME provides a unified framework enabling understanding of key properties of tumor evolution, as well as offering a foundation for evolution-informed therapeutic strategies.

## 2 Materials and methods

**Figure 1:**
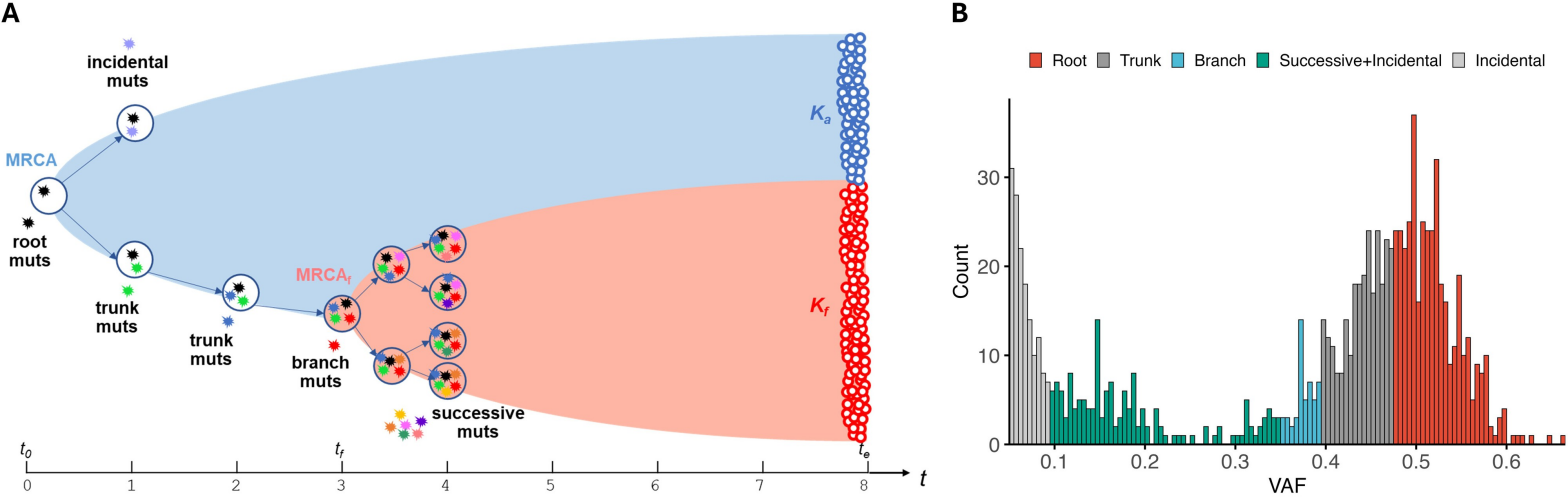
Schematic representation of TEATIME algorithm. (A) Tumor growth modeled as a discrete-time discrete-state Markov process. Cell divisions occur at a constant rate and at specific time points. Each division introduces mutations. The state of a cell is defined by its mutation composition. Asterisks with different colors represent mutations acquired at different time points. At time $t_{0}$, a single cell initiates the growth of a cell population via division. The growth progresses to the emergence of a subclone at time $t_{f}$, and continues until the point of sampling and sequencing at time $t_{e}$. Among cells with fitness similar to that of the initial cell, 1 division cycle occurs in one unit of time. Among cells with a higher fitness than the initial cell, >1 division cycle occurs in one unit of time. Blue and pink areas represent the expansions of the ancestral clone $K_{a}$ and the advantageous subclone $K_{f}$, respectively. (B) Decomposing the VAF distribution to different mutation groups.

We model cell division and mutation accumulation in a tumor as a discrete-time, discrete-state Markov process (Williams *et al.* 2018, Salichos *et al.* 2020, Ouellette and Awadalla 2022) (Fig. 1A). Starting with a single cell at time point $t_{0}$, its mitosis produces two daughter cells. Each daughter cell has a probability of dying before entering the next division cycle. Continued division of surviving cells over time leads to the formation of a population, with the initial cell at $t_{0}$ being the most recent common ancestor (${\mathrm{MRCA}}_{0}$). The growth of this population follows an exponential model, such that at a given time point ${t>t}_{0}$, the number of cells descended from the ${\mathrm{MRCA}}_{0}$ is


(1) $${N(t)=e}^{\lambda \beta (t-t_{0})}$$

where λβ is the cell proliferation rate (Supplementary Materials, available as supplementary data at *Bioinformatics* online). The unit of time is defined as the time between two consecutive cell divisions, so t can be interpreted as the number of generations. In a successful cell division, somatic mutations present in the parent cell are passed to the daughter cells and additional mutations are introduced at a rate μ, which is the number of mutations acquired per diploid genome per cell division. If all genetic or epigenetic alterations occurred after $t_{0}$ are selectively neutral, the cells grow at the same rate of λβ and form the ancestral clone $K_{a}$. If a cell acquires alterations at time $t_{f}$, which confers a growth advantage, it becomes the ${\mathrm{MRCA}}_{f}$ and proliferates at a rate λβ(1+s) with  s>0, forming a subclone $K_{f}$. The fitness advantage of $K_{f}$ over $K_{a}$ is quantified by the selection coefficient s, defined as the relative increase in net growth rates between the two populations. At the time point $t_{e}$ when a tumor is sampled for analysis, the total number of cells is N. The fraction of cells belonging to subclone $K_{f}$ among all tumor cells at $t_{e}$ is


(2) $$P=\frac{e^{\lambda \beta (1+s)(t_{e}-t_{f})}}{N}$$

The fraction of cells in $K_{f}\;$over those in $K_{a}$ is determined by the evolutionary parameters as


(3) $$ln(\frac{P}{1-P})=\lambda \beta (st_{e}-(1+s)t_{f})$$

Mutations acquired in the “foundation lineage” represent the evolutionary path from ${\mathrm{MRCA}}_{0}$ to ${\mathrm{MRCA}}_{f}$ (Fig. 1A). This lineage includes three classes of mutations depending on the time of acquisition. Root mutations, which preexist at $t_{0}$, are present in all tumor cells. Trunk mutations, acquired after $t_{0}\;$but before $t_{f}$, are present in all $K_{f}$ cells and some $K_{a}$ cells. For a trunk mutation acquired at time t, the fraction of tumor cells carrying it at $t_{e}$ is $f_{k}(t)$. The number of trunk mutations accumulated till time t is


(4) $$M_{k}(t)=\mu t=\frac{\mu }{\lambda \beta }[\ln(1-P)-\ln\left(f_{k}(t)-P\right)]$$

A total of μ branch mutations are acquired by the ${\mathrm{MRCA}}_{f}$ cell at time $t_{f}$. They are present in all $K_{f}$ cells but absent in $K_{a}$ cells. The fraction of cells carrying branch mutations at $t_{e}$ is $f_{h}=P$.

Outside the foundation lineage there are incidental mutations that are present in some $K_{a}$ cells but absent in $K_{f}$ cells. There are also successive mutations that are present in some $K_{f}$ cells but absent in $K_{a}$ cells. For a successive mutation acquired at time t, the fraction of cells carrying it at $t_{e}$ is


(5) $$f_{s}(t)=P\frac{1}{e^{\lambda \beta {(1+s)(t-t}_{f})}}$$

The above equations link the evolutionary process of a tumor to the fractions of cells carrying specific mutations at the time of sampling. These fractions can be inferred from VAFs reported by WES or WGS of bulk tumor cells (Ahmadinejad *et al.* 2022). For a heterozygous mutation in a diploid region, its VAF corresponds to half the fraction of cells carrying this mutation after adjusting for sample impurity. Leveraging these relationships, TEATIME transforms the estimation of evolutionary parameters to a task of decomposing a tumor’s mutational compositions (Fig. 1B). By maximizing the likelihood of observing the number of mutations in various categories and their VAF distributions, TEATIME infers maximum likelihood estimates for a set of parameters, including μ, s, $t_{f}$, and P (Supplementary Materials, available as supplementary data at *Bioinformatics* online).

## 3 Results

### 3.1 Evaluation of TEATIME performance

We used simulation data to evaluate the performance of TEATIME, MOBSTER (Caravagna *et al.* 2020), and TumE (Ouellette and Awadalla 2022), all of which operate in the subclonal evolution framework. The first simulated dataset, included with the MOBSTER package, comprises 150 synthetic tumor samples generated to follow a subclonal evolution trajectory based on the discrete-time discrete-state Markov process (Heide 2022). In these simulations, the following parameters were held constant, including *N* $=10^{8}$, μ=16, sequencing depth at 120× and a cell death rate of 0.2. Other parameters were varied across defined ranges, including $t_{f}\in [4,\;14]$, s∈[0.125, 1.625], and P∈[0, 0.97].

**Figure 2:**
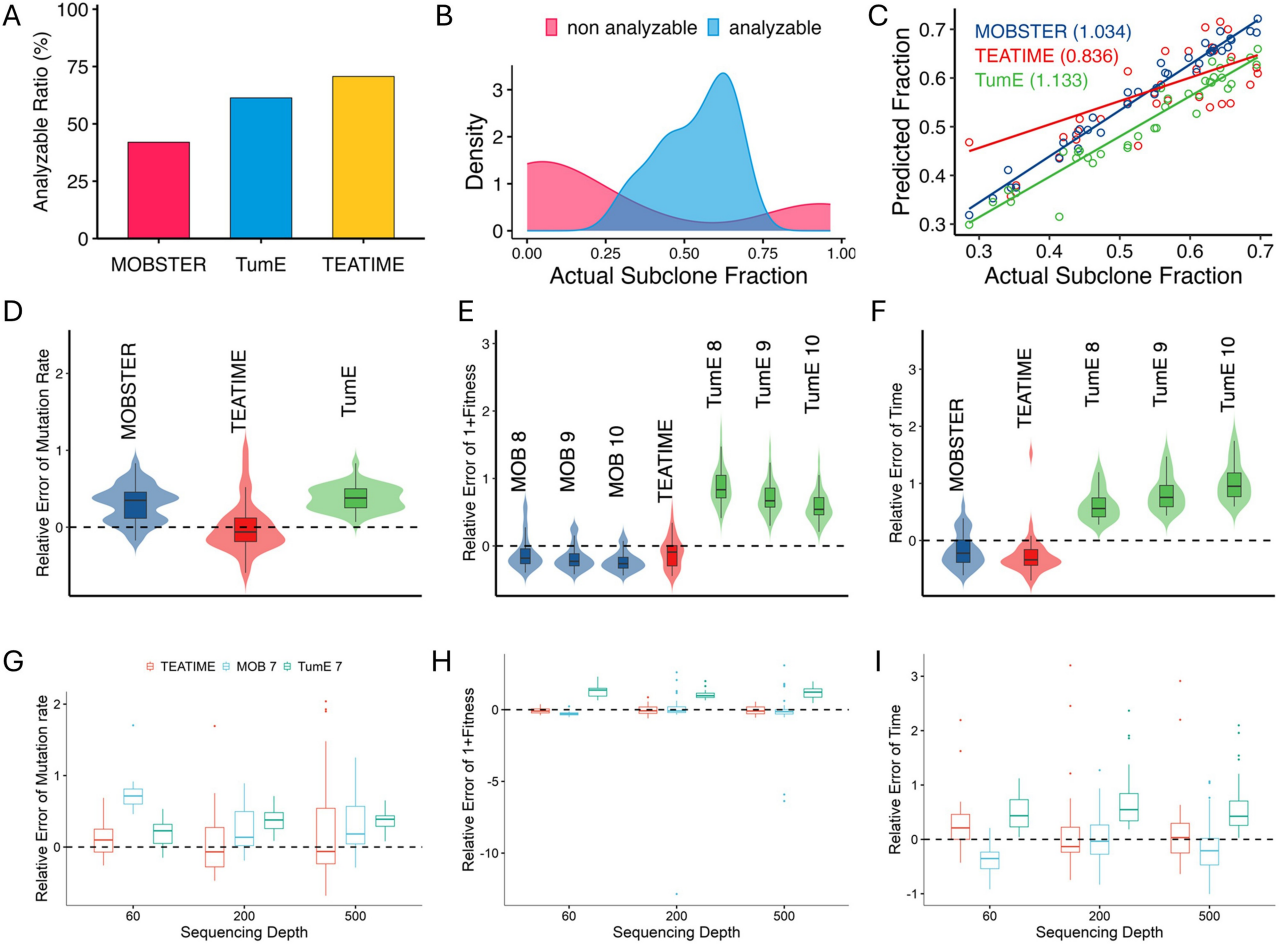
Comparison of the TEATIME, MOBSTER, and TumE on simulation datasets. (A) Proportion of analyzable tumors. (B) Density plot shows distributions of subclone fraction in tumors analyzable versus non-analyzable. (C) Scatter plot shows correlation between estimated and simulated subclone fractions. All regression slopes are highly significant (*P* < .001). (D–F) Violin plots show distributions of relative errors of estimated mutation rate (μ), fitness (s), and time of subclone emergence ($t_{f}$). A range of *N* values from 10^8^ to 10^10^ were provided to MOBSTER and TumE as input. Each configuration is annotated accordingly (e.g. MOB eight denotes MOBSTER with *N *= 10^8^). (G–I) comparison on datasets with varying sequencing depth and *N = *10^7^.

**Figure 3:**
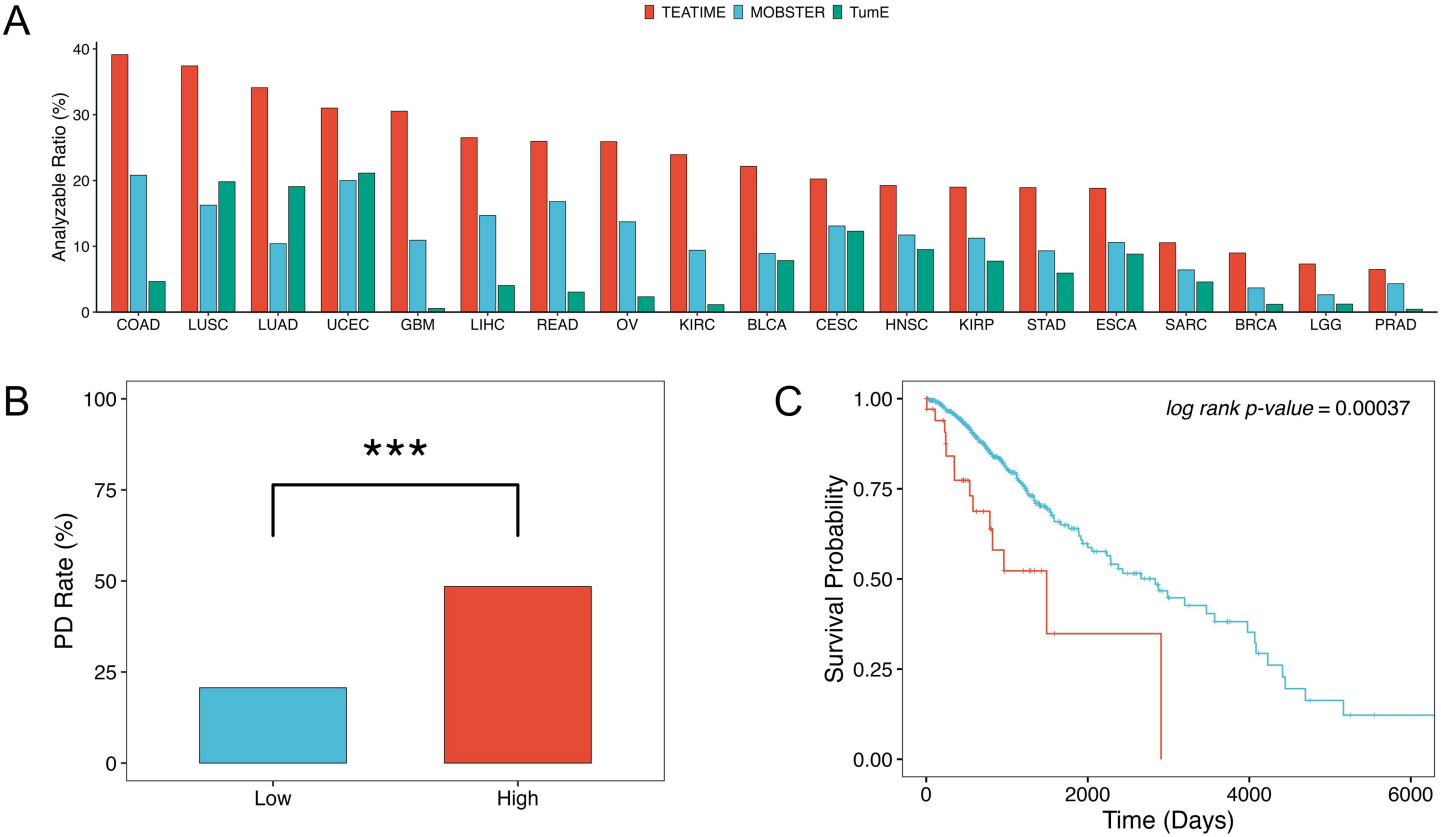
Fitness diversity across cancer types and its clinical relevance. (A) Fraction of analyzable tumors by cancer type. (B) Fraction of tumors exhibiting progressive disease (PD) after treatment, stratified by median fitness index θ (high vs. low). *** indicates *P* < .001. (C) Kaplan–Meier plot comparing overall survival between low-θ and high-θ groups (log-rank *P* < .001).

These tools did not produce results for all simulated tumors. We considered a tumor non-analyzable if the TEATIME or MOBSTER model failed to infer the complete set of evolutionary parameters. For TumE, tumors with fewer than five valid Monte Carlo estimates were deemed non-analyzable. MOBSTER was able to analyze only 42% of the simulated tumors, whereas TumE and TEATIME achieved success rates exceeding 60% and 70%, respectively (Fig. 2A). Notably, the non-analyzable tumors tended to have more extreme subclone fractions, either very low or very high, compared to the subclone fractions observed in analyzable tumors (Fig. 2B). To ensure a fair comparison, we restricted the subsequent analyses to the subset of 37 tumors that were successfully analyzed by all three methods. Since TumE produced a wide range of values for each parameter due to its Monte Carlo sampling approach (Fig. 3A and B, available as supplementary data at *Bioinformatics* online), we used the mean as the final estimation.

Despite having the lowest proportion of analyzable tumors, MOBSTER’s estimated subclone fractions showed an almost perfect correlation with the simulated values (Regression coefficient RC = 1.03, Fig. 2C). TEATIME and TumE also demonstrated high accuracy, with slightly lower but still very strong correlations (RC = 0.84 and 1.13, respectively). When evaluating additional parameters, including *μ*, *s* and $t_{f}$, TEATIME consistently outperformed the other two methods, achieving the lowest relative errors centered around zero (Fig. 2D–F). In contrast, MOBSTER tended to overestimate *μ* and underestimate *s* and $t_{f}$, whereas TumE systematically overestimated all three parameters (Fig. 2D–F). Because MOBSTER and TumE require *N* to calculate *s* or $t_{f}$, we examined the sensitivity of their estimations to different values of *N* (Fig. 3C and D, available as supplementary data at *Bioinformatics* online). Surprisingly, providing a value of *N* that deviated from the simulated ground truth sometime led to smaller errors.

**Figure 4:**
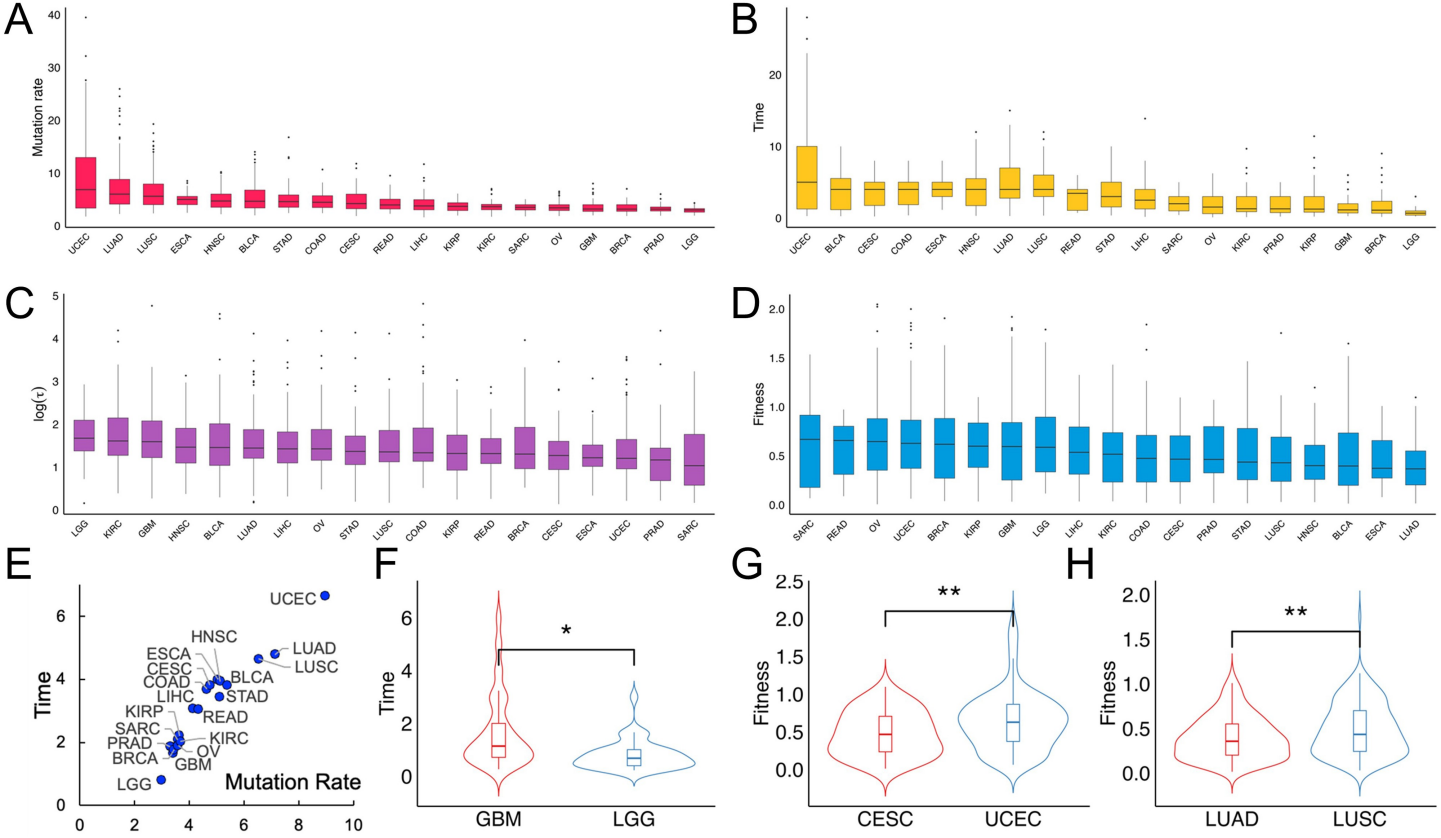
Pan-cancer distribution of inferred evolutionary parameters. (A–D) Boxplots show distributions of mutation rate (A), subclone emergence time (B), subclone expansion score (C), and subclone fitness (D) across cancer types. (E) Cancer types with higher mean mutation rates tend to exhibit later subclone emergence time. (F–H) Violin plots show distributions of inferred evolutionary parameters between cancer types in the same organ: brain (F), uterus (G), and lung (H).

**Figure 5:**
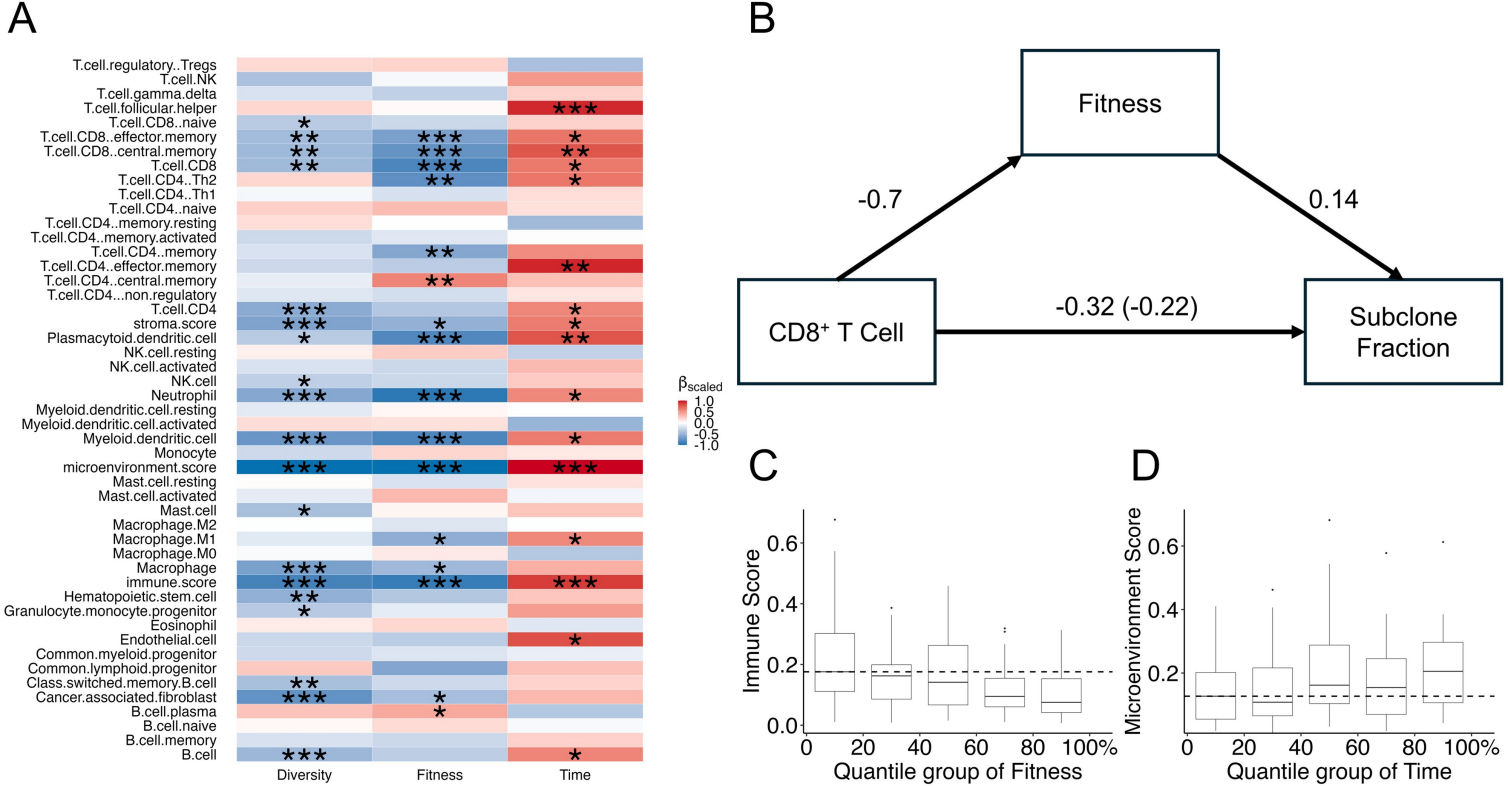
Association between evolutionary dynamics and the tumor microenvironment. (A) Heatmap of multivariable regression coefficients linking fitness diversity index θ, subclone fitness s, and subclone emergence time $t_{f}$ to immune cell abundances; asterisks denote FDR < 0.05 (*), <0.01 (**) or <0.001 (***). (B) Mediation model with CD8^+^ T cell abundance in microenvironment as the exposure, subclone fitness as the mediator, and subclone cellular fraction as the outcome (average causal mediation effect = –0.097, *P *= .002; average direct effect = –0.227, *P *= .042; total effect = –0.325, *P *= .002; proportion mediated = 30.5%, *P *= .004). (C) In LUSC, tumors with immune-hot microenvironment, as indicated by high immune score (C) and high microenvironment score (D), tend to harbor subclones with low fitness and late emergence time.

To complement the initial simulations where sequencing depth and final cell population size were fixed, we used the TEMULATOR (Heide 2022) to produce additional datasets using a series of d ranging from 60× to 1000× and *N* ranging from 10^6^ to 10^7^. Interestingly, increasing sequencing depth and providing true *N* values did not improve the estimations for MOBSTER and TumE (Fig. 2G–I, Figs 4 and 5, available as supplementary data at *Bioinformatics* online). In contrast, TEATIME produced parameter estimates with relative errors more tightly centered around zero, particularly at sequencing depths exceeding 100×. Because TumE outputs multiple values for each parameter, we plotted the 95% confidence ellipses of relative errors using all Monte Carlo estimates alongside TEATIME predictions. The ellipse area for TumE was markedly larger than that of TEATIME. Again, increasing sequencing depth did not substantially reduce the ellipse area for TumE, in contrast to TEATIME, where higher depth led to more accurate estimates (Figs 4D and 5D, available as supplementary data at *Bioinformatics* online).

These results collectively demonstrated that TEATIME outperformed MOBSTER and TumE by producing minimal expected relative error in estimating *μ*, *s*, and $t_{f}$ without requiring prior information on *N*.

### 3.2 Pan-cancer analysis of tumor evolution

**Figure 6:**
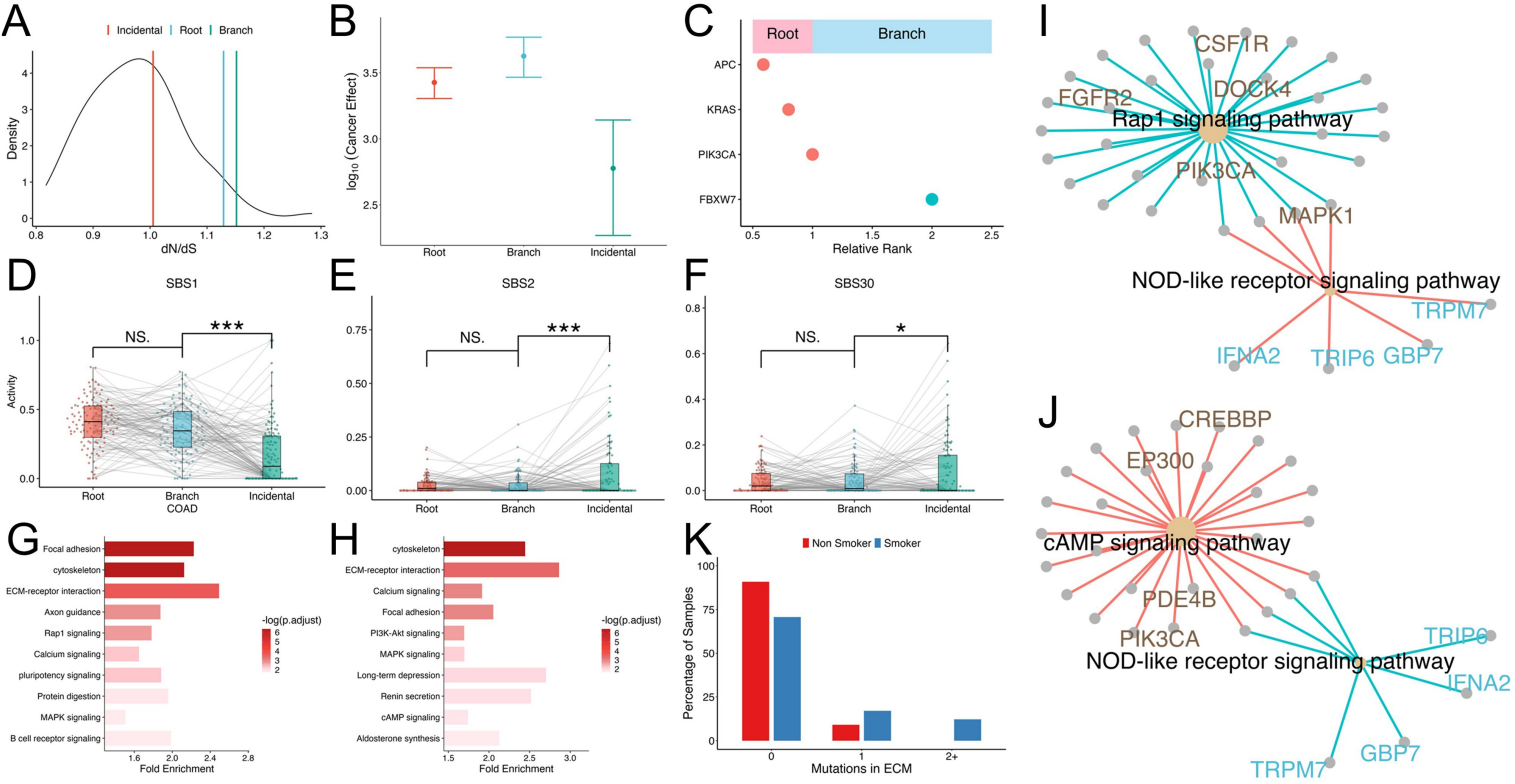
Characteristics of mutations acquired at various time points. (A) dN/dS ratios for root, branch, and incidental mutation; the density curve represents background dN/dS ratios calculated from randomly sampled incidental mutations. (B) Cancer effect size with 95% confidence interval for root, branch and incidental mutations. (C) Genes ranked by the ratio of their mutation frequencies in root versus branch categories. (D–F) Mutational signature decomposition for root, branch and incidental mutations, illustrating distinct signature profiles across temporal categories. * (*P*<.05); ** (*P*<.01); *** (*P*<.001) (G and H) Top ten signaling pathways enriched among root-mutated genes (G) and branch-mutated genes (H). (I and J) Pathway network demonstrating that root mutations in key pathways increase the likelihood of downstream branch mutations. (K) Correlation between patient smoking status and root‐mutation burden in extracellular matrix (ECM) related pathways (*P* < .05).

We extracted WES data of 8935 primary tumors representing 33 cancer types from the TCGA study (Grossman *et al.* 2016). For each tumor, we used SNVs located in diploid genomic regions to infer evolutionary parameters. Because <8% of these tumors were analyzable by MOBSTER and TumE (Fig. 6, available as supplementary data at *Bioinformatics* online), we focused on TEATIME analysis results.

TEATIME produced evolution estimators for 1628 (18%) tumors (Fig. 3A, Table 1, available as supplementary data at *Bioinformatics* online). The proportion of analyzable tumors varied substantially across cancer types. For example, in colon adenocarcinoma (COAD), approximately 40% of tumors yielded results, whereas in acute myeloid leukemia (AML), only a single case was analyzable. Because TEATIME assumes the presence of two subpopulations with distinct fitness levels, tumors deemed non-analyzable likely exhibit a more homogeneous fitness landscape compared to those that are analyzable. This hypothesis aligns with the stem cell model of AML, in which a small population of cancer stem cells continuously gives rise to progenitor cells that differentiate into tumor cells without forming an apparent subclonal structure (Magee *et al*. 2012, Chu *et al.* 2024). To ensure sufficient statistical power, we limited the subsequent analysis to 19 tumor types that had at least 20 analyzable tumors.

#### 3.2.1 Fitness diversity is a new dimension of intra-tumor heterogeneity

The extent of mixing between $K_{a}$ and $K_{f}$ clones in a tumor reflects the degree of heterogeneity among competing cell populations. To quantify this, we define a fitness diversity index θ based on Shannon entropy, which captures the imbalance between cell populations with distinct fitness levels (Supplementary Materials, available as supplementary data at *Bioinformatics* online). The θ ranges from 0 to 1, with 0 indicating a tumor composed entirely of $K_{a}$ or $K_{f}$ clones (i.e. no diversity), and 1 indicating a perfectly balanced mixture of $K_{a}$ and $K_{f}$ clones. For non-analyzable tumors, we assume they are dominated by either $K_{a}$ or $K_{f}$, and assign θ=0. We found that θ is a novel prognostic marker. In low-grade glioma (LGG), tumors with high θ were associated with an increased risk of poor response to treatment (*P *= 8 × 10^−5^, Fig. 3B) and shorter overall survival (*P *= 4 × 10^−4^, Fig. 3C). This association remained significant even after adjusting for age, sex and *IDH1* mutation status (Cox regression *P *= 9 × 10^−4^), suggesting θ is an independent prognostic marker.

### 3.3 Pan-cancer subclonal evolution characteristics

Mutation rates varied across cancer types. On average, the mutation rate of LGG tumors was the lowest (mean *μ* = 2.9 per exome per generation), while uterine corpus endometrial carcinoma (UCEC) was the highest (mean *μ* = 8.9 per exome per generation, Fig. 4A) (Lawrence *et al.* 2013). In most tumor types, derived subclones emerged early. The mean $t_{f}$ ranged from 1 generation after $t_{0}$ in LGG to 7 generations in UCEC, where a higher value of $t_{f}$ indicates a later emergence of the subclone (Fig. 4B). This observation is consistent with previous studies reporting that aggressive subclones, such as those associated with metastasis or treatment resistance, often arise near the onset of tumorigenesis (Röcken 2010, Hosseini *et al.* 2016, Hu *et al.* 2019). To quantify the duration of subclone expansion relative to its emergence, we calculated a subclone expansion score $\tau =t_{e}/t_{f}$. This score ranged from a mean of 4.4 in esophageal carcinoma (ESCA) to 7.9 in bladder urothelial carcinoma (BLCA, Fig. 4C), suggesting that most subclones undergo extended growth prior to clinical detection. Meanwhile, the estimated selection coefficients s were moderate across all tumors, with none exceeding 2.04 (Fig. 4D). These findings indicate that despite the sustained expansion of subclones, their modest fitness advantages constrain rapid or explosive growth.

Interestingly, we found that in tumor types with high mutation rates, derived subclones tended to emerge relatively late (Fig. 4E). These tumors, including UCEC, lung adenocarcinoma (LUAD), lung squamous cell carcinoma (LUSC), BLCA, and ESCA, are known to exhibit high mutational burdens, often resulting from prolonged exposure to inflammation, environmental insults, or other factors during extended precancerous stages (Chalmers *et al.* 2017, Jamal-Hanjani *et al.* 2017, Sha *et al.* 2020). The delayed emergence of subclones in these contexts suggests that while mutational accumulation occurs early and broadly, the selective advantage required for subclonal expansion may only arise later, possibly due to microenvironmental shifts or late-arising driver mutations.

Cancers arising in the same organ but differing in histological type often display distinct clinical features. For example, LGG typically presents at a younger age than GBM (Lin *et al.* 2020), and LUSC is associated with larger tumor size and poorer outcomes compared to LUAD (Anusewicz *et al*. 2020, Wang *et al.* 2022, Wu *et al.* 2023). Consistent with these clinical distinctions, we observed significant differences in their evolutionary dynamics. In brain tumors, LGG exhibited an earlier subclone emergence time than glioblastoma multiforme (GBM, *P *= .02, Fig. 4F). In uterine tumors, subclone fitness was higher in UCEC compared to cervical squamous cell carcinoma and endocervical adenocarcinoma (CESC, *P *= .0044, Fig. 4G). A similar trend was observed in lung cancers, where subclones in LUSC exhibited markedly higher fitness than those in LUAD (*P *= .002, Fig. 4H). These findings warrant further investigation into how evolutionary differences relate to underlying clinical features.

### 3.4 Association of immune infiltration with tumor heterogeneity and evolutionary trajectory

In addition to intratumor competition, the immune microenvironment may also impose selective pressures to shape a tumor’s evolutionary trajectory. To investigate this, we obtained pre-computed tumor-infiltrating immune cell abundances in TCGA tumors using three tools, including TIMER (Li *et al.* 2020), xCell (Aran *et al.* 2017), and CIBERSORT (Newman *et al.* 2015). We then performed a pan-cancer multivariate regression analysis to assess associations between immune cell infiltration scores and evolutionary parameters (Supplementary Materials, available as supplementary data at *Bioinformatics* online). Our analysis revealed that both the fitness diversity index θ and subclone selection coefficient s were consistently and negatively associated with the infiltration levels of multiple immune cell types, while subclone emergence time $t_{f}$ showed positive associations (FDR < 0.05, Fig 5A). These patterns suggest that in tumors with abundant immune infiltration, subclones tend to emerge later and exhibit lower selection advantages, resulting in reduced fitness diversity. While previous studies have shown that “immune-hot” microenvironments can constrain intratumor genetic heterogeneity (Morris *et al.* 2016, Şenbabaoğlu *et al.* 2016, Milo *et al.* 2018, Lin *et al.* 2020, Ge *et al.* 2022, Denisenko *et al.* 2024, Mo *et al.* 2024), our results offer additional insight from an evolutionary perspective.

We next sought to establish the causal relationship among these parameters via mediation analysis, in which microenvironment factors served as exposures, evolutionary parameters are mediators, and subclone fraction as the outcome. The results showed that 20 immune-cell subsets significantly influenced the subclone fitness and emergence time, which in turn affected the subclone fraction (Table 2, available as supplementary data at *Bioinformatics* online). For example, CD8^+^ T cells were found to suppress subclone expansion, with 30.5% of this effect mediated through their negative impact on subclone fitness (*P* = .004; Fig. 5B). Tumor-type specific models revealed the same patterns (Table 3, available as supplementary data at *Bioinformatics* online, Fig. 5C and D), reinforcing the conclusion that immune surveillance imposes a universal constraint on subclonal outgrowth.

### 3.5 Characteristics of root and branch mutations

**Figure 7:**
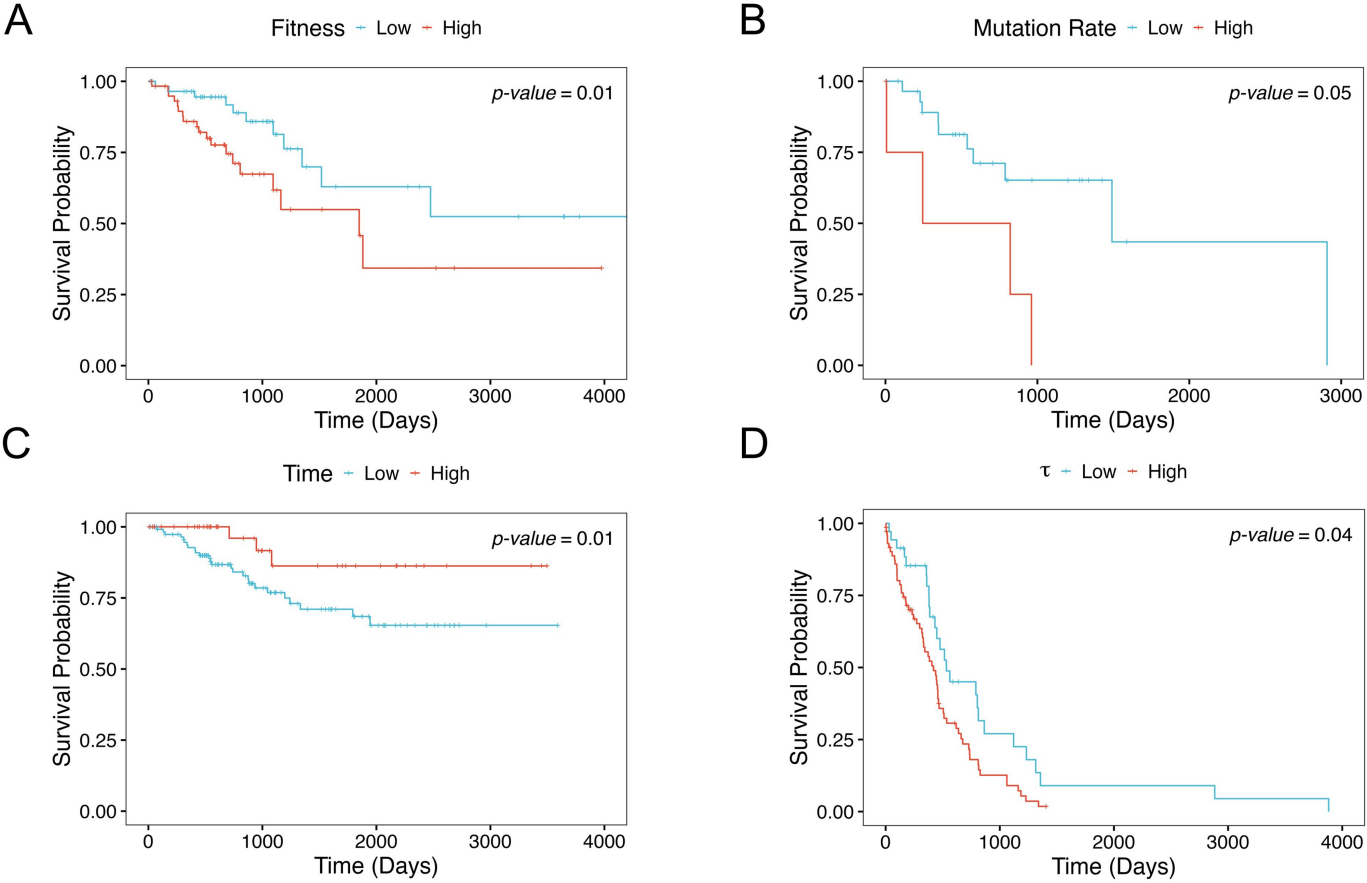
Association of evolutionary parameters with clinical features. Kaplan–Meier plots compare survival rates between two tumor groups based on various evolutionary parameters. The thresholds used to stratify tumors were determined based on the best *P* values. (A) COAD tumors stratified into low versus high fitness groups. (B) groups LGG tumors stratified into low versus high mutation rate groups. (C) UCEC tumors stratified into low (early emerging) versus high (late emerging) subclone emergence time. (D) GBM tumors stratified into high versus low τ groups.

In the TEATIME model, ${\mathrm{MRCA}}_{0}$ acquires growth advantage over normal cells, and ${\mathrm{MRCA}}_{f}$ gains a further advantage over the ancestral clone. If this model holds true, we expect root mutations in ${\mathrm{MRCA}}_{0}$ and branch mutations in ${\mathrm{MRCA}}_{f}$ to be enriched with drivers. To test this hypothesis, we used the dNdScv package (Martincorena *et al.* 2017) to computed dN/dS ratios for root, branch, and incidental mutations aggregated over all tumors of the same type. The background distribution was generated across 100 iterations, each randomly sampling one incidental mutation from a tumor, pooling them over all tumors, and calculating the dN/dS ratio (Materials, available as supplementary data at *Bioinformatics* online). In multiple cancer types, dN/dS ratios of root and branch mutations were significantly elevated compared to incidental mutations, indicating evidence of positive selection (Fig. 7, available as supplementary data at *Bioinformatics* online). Using COAD as an example, branch mutations exhibited the highest dN/dS ratio (1.15), followed by root mutations (1.12), both of which were significantly higher than incidental mutations (1.0; *t*-test *P *= 1.56×10^−37^ and 8.08×10^−33^, respectively, Fig. 6A). To account for the influence of gene-specific mutation rates on dN/dS ratio, we further calculated the cancer effect size of root, branch, and incidental mutations in each cancer type using the cancereffectsizeR package (Cannataro *et al.* 2018). Consistent with the dN/dS analysis, effect size estimates followed the same pattern: branch and root mutations exhibited significantly larger effect sizes than Incidental mutations (log-scale effect size = 3.62, 3.42 and 2.77, respectively, *P *< .05, Fig. 6B, Fig. 8, available as supplementary data at *Bioinformatics* online). We observed similar trends in BLCA, LUAD, LIHC, BRCA, HNSC, KIRC and CESC tumors (Figs 7 and 8, available as supplementary data at *Bioinformatics* online).

We next compared mutational signatures of root, branch, and incidental mutations. Using COAD as an example, we identified three mutational signatures with distinct activity profiles across these groups (Fig. 6D–F). The first signature SBS1 showed high activity in both root and branch mutations but declined significantly in incidental mutations (FDR = 1.12×10^−7^). In contrast, SBS2 and SBS30 were more active in incidental mutations than in root and branch mutations (FDR = 0.003 and 0.07, respectively). We further examined the known etiologies of these signatures. SBS1 is a clock-like signature associated with the spontaneous deamination of 5-methylcytosine. The high activity of this signature in root and branch mutations imply that mutation accumulations in the early stages of tumorigenesis is largely due to aging processes. However, as tumors progress and undergo genome-wide hypomethylation, the pool of 5-mC substrates available for deamination decrease (Yegnasubramanian *et al.* 2008, Yan *et al.* 2023), potentially explaining the reduced SBS1 activity in incidental mutations. In fact, we observed a late-stage decline in SBS1 activity across five cancer types (Fig. 9, available as supplementary data at *Bioinformatics* online), consistent with the known timing of hypomethylation as a late event in these cancer types. SBS2 and SBS30 are both associated with perturbations in base excision repair, but through distinct processes (Petljak *et al.* 2019, 2022, Shen *et al.* 2020, Butler and Banday 2023). SBS2 reflects APOBEC3 activity, while SBS30 is linked to inactivation of NTHL1. The increased activity of these signatures in incidental mutations suggests that DNA repair deficiencies become more pronounced at later stages of tumor evolution.

#### 3.5.1 Temporal relationships

To better understand the evolutionary progression leading to the emergence of the derived subclone, we examined the relationships between root and branch mutations using COAD as an illustrative example. We began by calculating the frequency of mutations in each gene classified as root or branch, and ranked genes based on the ratio of root-vs-branch frequency (Fig. 6C). This simple metric revealed that *APC* was the most frequently mutated gene in the root, followed by *KRAS* and *PIK3CA*. In contrast, *FBXW7* was most commonly mutated in the branch. This pattern aligns with the established sequence of genetic alterations in colorectal cancer progression: *APC* → *KRAS* → *PIK3CA* → *FBXW7* (Fearon and Vogelstein 1990, Smith *et al.* 2002, Gerstung *et al.* 2011), and reinforces the role of *APC* as an early driver in colorectal carcinogenesis.

We then categorized genes into root or branch groups based on whether they were most frequently mutated in ${\mathrm{MRCA}}_{0}$ or ${\mathrm{MRCA}}_{f}$, respectively. Based on functional annotations from the KEGG database, we performed overrepresentation tests on root-mutated genes. The results revealed that multiple pathways were differentially perturbed at this evolutionary stage (Fig. 6G, Fig. 10A, available as supplementary data at *Bioinformatics* online). In COAD, we observed significant enrichment in canonical cancer-related pathways, including PI3K–AKT signaling and MAPK signaling, as well as several pathways related to the tumor microenvironment, such as extracellular matrix (ECM) organization and focal adhesion. Interestingly, branch-mutated genes were also enriched in a largely overlapping set of pathways (Fig. 6H, Fig. 10B, available as supplementary data at *Bioinformatics* online), suggesting that both early and late drivers tend to converge on core oncogenic and microenvironmental processes. In contrast, incidental mutations showed no significant enrichment in any pathway, further supporting their presumed lack of functional relevance (Fig. 10C, available as supplementary data at *Bioinformatics* online).

Next, we explored temporal dependencies between root and branch mutations. For each tumor, we annotated mutations based on the functional pathways of their associated genes. We then performed linear regression to assess whether root mutations predispose specific pathways to be mutated in the branch. In CESC, we found that mutations in Rap1 signaling and cAMP signaling pathways in ${\mathrm{MRCA}}_{0}$ increased the likelihood of the NOD-like receptor signaling pathway being mutated in ${\mathrm{MRCA}}_{f}$. Such influence might be due to connections between these pathways via shared genes (Fig. 6I and J). In LUAD, mutations in ECM–receptor interaction pathway in ${\mathrm{MRCA}}_{0}\;$predisposed to mutations in the NOD-like receptor signaling pathways in ${\mathrm{MRCA}}_{f}$. Although these two pathways do not share genes, they are both involved in tumor–microenvironment interactions, suggesting functional interplay (Wong and Rustgi 2013, Yue 2014, Hassanein *et al*. 2021, Tan *et al.* 2021). Notably, we found that smokers exhibited significantly more ECM-related mutations compared to non-smokers (*P* = .047, Fig. 6K), suggesting smoking may act as an upstream factor, reshaping the tumor microenvironment to which evolving cancer clones adapt.

### 3.6 Association with clinical phenotypes

Lastly, we found that evolutionary parameters have prognostic power in many cancer types. In COAD, elevated subclone fitness predicted worse prognosis (FDR = 0.1, Fig. 7B). Several other cancer types also showed trends indicative of prognostic relevance at nominal *P *< .05, although FDR corrected for multiple comparison did not reach statistical significance. In LGG, higher mutation rate was linked to poorer overall survival (Cox regression *P *= .047, Fig. 7A). In UCEC, earlier emergence of derived subclone conferred adverse outcome (Cox regression *P *= .013, Fig. 7C). In GBM, increased subclone expansion score τ was associated with reduced survival (Cox regression *P* = .042, Fig. 7D). Pan-cancer analysis further confirms that the emergence timing of subclone carries prognostic relevance, specifically that early arising aggressive subclones are linked with adverse outcomes (*P* = .027, Fig. 11, available as supplementary data at *Bioinformatics* online). These results demonstrate that various aspects of tumor evolutionary dynamics carry prognostic information across cancer types.

### 3.7 Validation of TEATIME inferences using paired primary-metastatic tumors

Although direct validation of TEATIME inference in clinically sequenced tumors is challenging, we leveraged paired primary–metastatic samples to indirectly assess the model’s predictions (Supplementary Materials, available as supplementary data at *Bioinformatics* online). The rationale is that if seeding clones correspond to early evolutionary events inferred by TEATIME, their associated metastases should exhibit fewer pre-dissemination mutation and more post-dissemination mutations than those seeded from later evolutionary events. We analyzed primary-metastatic pairs sequenced in the Metastatic Breast Cancer Project (Wagle *et al.* 2017). The results supported this expectation in both tumors with multiple seeding events and those with a single seeding event (Fig. 12, available as supplementary data at *Bioinformatics* online).

## 4 Discussion

Understanding the evolutionary dynamics of tumors is essential for uncovering the mechanisms that drive tumor heterogeneity, progression, and therapeutic resistance. In this study, we present TEATIME, a novel computational framework that infers mutation rate, subclone fitness, and subclone emergence time from bulk sequencing data. In benchmarking analyses, TEATIME consistently outperformed existing tools in both accuracy and robustness across a wide range of tumor sizes and sequencing depths. Importantly, TEATIME yielded 2- to 37-folds of increase on the number of analyzable tumors across various cancer types, underscoring its utility for real-world clinical genomics applications.

By introducing fitness diversity as a novel measure of intratumor heterogeneity, we uncovered a universal constraint imposed by immune-inflamed microenvironments: Tumors with greater immune infiltration across multiple effector and regulatory cell types exhibit less fitness diversity. Mediation analyses reveal that this constraint operates indirectly, through reductions in subclone fitness and delayed emergence. These results integrate and extend previous reports, demonstrating that immune surveillance imposes selective pressure on tumor evolutionary trajectories by modulating both the fitness landscape and temporal ordering of emerging clones.

By comparing root, branch and incidental mutations we uncover a temporal inflection in subclone emergence. Branch mutations are under intensified positive selection and enriched in cell adhesion, ECM remodeling, and inflammatory signaling pathways. Mutational signature analysis shows that SBS2 activity increases after the onset of the more fit derived subclones while the clock-like SBS1 signature declines. These coordinated changes in selection pressure, pathway engagement, and mutational signatures define the consequences of the emergence of subclone outgrowth. These results are consistent with the Big Bang model of tumorigenesis which posits that most driver mutations occur early followed by rapid clonal expansion and later genomic stability.

Importantly, the evolutionary parameters inferred by TEATIME carry independent prognostic value. In multivariate survival models encompassing diverse cancer types, higher subclone fitness and earlier emergence both predict poorer overall survival. Thus, TEATIME not only reconstructs past evolutionary trajectories but also forecasts clinical outcomes We foresee several immediate applications. First, fitness diversity may serve as a novel prognostic biomarker, as high fitness diversity could imply suppressed immunity (Fig. 5A) and could identify patients at greater risk of rapid progression or relapse (Fig. 3B and C). Second, the timing of subclone emergence can help distinguish clonal from subclonal driver events, while the subclonal cell fraction may inform treatment outcome predictions for therapies targeting subclonal versus clonal drivers. Third, for treatment stratification, patients harboring early-emerging, high-fitness subclones may benefit from combinatorial or adaptive therapeutic strategies rather than standard regimens. Fourth, in treatment monitoring and adaptive intervention, serial inference of subclonal parameters could enable early detection of emerging resistant populations and guide timely therapy adjustments. Collectively, these metrics can be integrated with other biomarkers to train predictive models, introducing an evolutionary dimension to tumor characterization and complementing established molecular and immunological indicators to refine prognosis and therapeutic decision-making.

TEATIME is built on several assumptions to ensure computational tractability and applicability to bulk sequencing data, including diploidy, infinite-sites model, exponential growth, existence of a single derived advantageous subclone, and comprehensive sequencing of high-frequency mutations acquired in the early foundation lineage. Violation of these assumptions will reduce the accuracy of the estimations. While these assumptions are common among existing methods in this area, we provide detailed discussion of the rationale, limitations, and potential biases associated with these assumptions to help users better understand the scope and applicability of these methods.

- Diploidy: Using SNAs from diploid regions allows the VAF to be interpreted as half of the fraction of cancer cells carrying these mutations. This assumption is also essential for clock-like mutational processes of SNAs, as diploid regions are less likely to have undergone large-scale genomic alterations that confound temporal inference (Manders *et al.* 2021). As a preprocessing step, TEATIME excludes genomic regions affected by CNAs to ensure analysis is restricted to diploid regions. However, when too few SNAs remain after filtering, clustering and estimation of subclonal parameters become unstable or infeasible, limiting the model’s applicability to highly rearranged tumors.
- Infinite-sites model: TEATIME adopts the infinite-sites model, which assumes that each genomic site mutates at most once during tumor evolution. This assumption simplifies phylogenetic reconstruction by enforcing a consistent and irreversible relationship among mutations, allowing VAFs to serve as markers of ancestry. However, when this assumption is violated, such as in hypermutated tumors, ignoring recurrent or back mutations can lead to underestimation of the overall mutation rate, earlier inferred acquisition time, and distortion of the other evolutionary parameter estimates. To minimize this potential confounding effect, we excluded hypermutated tumors from our analysis of the TCGA samples.
- Exponential growth model: TEATIME assumes that tumor cell growth is unconstrained and that all cell divisions are symmetric, corresponding to an exponential growth model. Under this assumption, the cell proliferation rate remains constant over time and across space [λβ in Equation (1)]. In tumors subject to spatial constraints or resource limitations, the proliferation rate inferred under this model will be an overestimation. We acknowledge that more sophisticated frameworks, such as sub-exponential or boundary-driven (2D or 3D) models, allow spatially and temporally varying proliferation rates. However, these approaches require explicit specification of spatiotemporal growth functions with additional parameters, substantially increasing computational complexity, and any mis-specification could introduce additional biases.
- A single derived subclone: Bulk DNA sequencing of a single tumor sample at a moderate depth (e.g. 50x–120x in TCGA) provides limited resolution to distinguish mutation clusters with similar VAFs (Ahmadinejad *et al*. 2022) (ΔVAF<0.05). This constraint underlies our assumption of only two competing cell populations, one ancestral clone and one derived subclone. In the current framework, reconstructing evolutionary dynamics of these two populations requires identifying at least five mutation clusters, corresponding to root, incidental, trunk, branch, and successive mutations, respectively. For a subclone that emerges later or experience strong positive selection, additional trunk and successive mutation clusters would need to be detected. Introducing more subclones would further increase the number of required clusters, pushing the limits of reliable inference from bulk sequencing data. Therefore, we allow only one derived subclone, which serves as a proxy for the aggregate behavior of all positively selected subclones. The inferred emergence time and fitness thus represent the average properties of these subclones.

Although several methods relax these constraints to reconstruct tumor phylogenies (Bonizzoni *et al.* 2019, Lu 2025), they do not estimate quantitative evolutionary parameters, which is the primary aim of TEATIME. Incorporating large-scale genomic alterations, SNAs in non-diploid regions, adjustments for recurrent or back mutations, non-exponential growth models, and multiple advantageous subclones into high-resolution tumor phylogeny reconstruction represents an important direction for future research, which we are actively pursuing.

Beyond these modeling constraints, all tools for reconstructing tumor evolutionary history require a sufficiently large number of mutations to ensure robust performance. Therefore, we do not recommend using these tools to analyze tumors with low mutation burden or those assessed using targeted sequencing. In fact, TEATIME failed to converge for approximately 80% of TCGA tumors due to these constraints. We further note that MOBSTER was developed for WGS, which may limit its performance on WES datasets. Additionally, because bulk sequenced biopsies capture only a limited region of the tumor, the identified subclones may reflect spatially localized populations rather than the complete evolutionary landscape. Because metastatic tumors are often seeded by multiple cells, they are not suitable for TEATIME analysis. Extending the framework to model multiple coexisting subclones, spatial heterogeneity, and non-exponential growth dynamics, as well as incorporating longitudinal or single cell sequencing data, will broaden its applicability.

To strengthen reproducibility, we provide an example workflow in the Supplementary Materials, available as supplementary data at *Bioinformatics* online and in the TEAMTIME GitHub site.

## Supplementary Material

btag127_Supplementary_Data

## Data Availability

The datasets were derived from sources in the public domain GDC Data Portal (https://portal.gdc.cancer.gov/).
