# Supplementary material for: Competing subclones and fitness diversity shape tumor evolution across cancer types
Source: Bioinformatics. 2026 Mar 13;42(3):btag127. doi: 10.1093/bioinformatics/btag127 (PMC13025073; doi:10.1093/bioinformatics/btag127)
Supplement: btag127_Supplementary_Data [file btag127_supplementary_data.pdf]

SUPPLEMENTARY MATERIALS

Cell proliferation parameters

The parameter λ controls the cell birth rate. By setting $\lambda = \ln 2$, it indicates that the division of a parent cell produces two daughter cells. The parameter $\beta = 1 + {}^{\ln \varphi}/_{\lambda}$, where φ defined as cell birth rate – cell death rate. Users can specify the value of β. By default, we set $\beta = 0.9$.

Input data and preprocessing

The input data for TEATIME has three columns: the read count of the wildtype allele, the read count of the mutant allele, and the ploidy of the genomic region harboring the mutation. Only heterozygous mutations located in diploid regions are used. To estimate the sample impurity, VAFs are clustered using the MAGOS program and the mean VAF is calculated for each cluster. The highest mean VAF φ provides an estimate of the sample purity $\rho = 2\varphi$. To adjust for sample impurity, the VAF of each mutation is adjusted to $VAF(2-\rho)/(2VAF(1-\rho)+\rho)$.

Simulations

The performance of TEATIME and other methods was evaluated using two sets of simulated data. The first dataset is part of the MOBSTER package[1], consisting of 150 synthetic tumors that follow various evolutionary trajectories. While all tumors contain 10^8 cancerous cells at the time of sampling, the other evolutionary parameters used to simulate tumor growth vary in a broad range, including $t_f \in [4, 14]$, $s \in [0.125, 1.625]$ and $P \in [0, 0.97]$.Some of these tumors are monoclonal neutral, containing no subclones, while others include one subclone. All simulated samples consist of 100% cancerous cells and are sequenced at a depth of 120×.

The second dataset was generated using TEMULATOR[2], where we varied the sequencing depth from 60× to 1000× and varied N from 10^6 to 10^7, and $t_f \in [4, 14]$, $s \in [0.125, 1.625]$ and $P \in [0, 0.92]$. The purity is 100% for simplicity. For each set of parameter values, 150 synthetic tumors were simulated. A total of 2,100 tumor samples were simulated. Each sample contains at least 1000 mutations.

TCGA data: We downloaded the mc3.v0.2.8.PUBLIC.maf.gz file from the Genomic Data Commons data portal[3], which contained somatic mutations in 10,295 tumors of 33 cancer types from TCGA PanCanAtlas mapped to the human genome build hg37. After removing hyper- and and hypo-mutated tumors, defined as the those with the total number of mutations outside the 1.5 interquartile range[4] , and removing relapsed and metastatic tumors, 8,935 primary tumors of 33 cancer types were retained. For each tumor, clinical information, CNVs, and annotated somatic mutations from WES were downloaded. To ensure sufficient statistical power, we restricted downstream analyses to cancer types with at least 20 predictable samples, resulting in 19 tumor types.

Quantification of intra-tumor heterogeneity

To quantify intra-tumor heterogeneity, we computed the fitness diversity index (θ) as the Shannon entropy of the cell population distribution. Let P denote the proportion of K_f cells, we then calculated

$$\theta = Pln(P) + (1 - P)\ln(1 - P)$$

Statistical tests of associations

Cox proportional hazards model was used to test the association between evolutionary parameters and patient overall survival. Covariates included age, gender, tumor stage, mutation load, presence of nonsynonymous mutations in driver genes if applicable. For each cancer type, driver genes were identified using the GUST program[4], not annotated as passenger genes in the Cancer Gene Consensus[5]. Presence of nonsynonymous mutations in oncogenes in the corresponding cancer type was coded as 1 and 0 otherwise. The coxph() function in the R/survival package was used for this analysis. For Kaplan–Meier visualization, tumor stratification thresholds were defined using optimal cut points identified by cut_survpoint(). Logistic Regression was used to test the association between evolutionary parameter and cancer type/Treatment Resistance, Covariate adjustment was performed in the same manner

as in the Cox proportional hazards models. For Treatment Resistance, Progressive disease (PD) in treatment outcome was treated as a binary outcome (PD = 1, non-PD = 0).

Linear regression was used to test the association between evolutionary parameter and immune infiltration, Models for the pan-cancer cohort included age and cancer type as covariates, whereas tumor specific analyses also incorporated gender, tumor stage and mutation load if applicable. P-values were adjusted using the Benjamini–Hochberg (BH) method.

Mediation tests in immune infiltration:

In the mediation test, microenvironment factors served as exposures, evolutionary parameters are mediators, and subclone fraction as the outcome. R mediation package is used for estimating indirect effect[6].

Selection intensity and dN/dS ratio Calculation

To evaluate dN/dS ratio, we applied the dNdScv R package[7]. We consider three locations: Root mutations, Branch mutations, and Incidental mutations. To construct a background distribution, we picked a random incidental mutation per tumor as the seed and included additional incidental mutations with VAFs ±0.01 of that seed, aggregated mutations over all tumors, computed dN/dS ratio and repeated for 100 iterations. A one-sample t-test was performed to evaluate whether the Root, Branch dN/dS ratios were significantly higher than the background dN/dS, estimated from randomly selected Incidental mutation. For Root mutations, Branch mutations, and Incidental mutations, we also calculated cancer effect size using the cancereffectsizeR package[8].

Mutational signature extraction and statistical test

Following mutational signature analysis practices[9], we extract mutational signatures at root, branch and incidental evolutionary stages by excluded SBS signatures considered biologically implausible or irrelevant for each cancer type[10,11]. We then applied the MutationalPatterns

package to extract mutational signatures at each location using a curated subset of cancer type–specific SBS signatures[12].

After signature extraction, we performed paired t-tests to compare the relative contribution of each SBS signature between root and branch, and between branch and incidental mutations. P-values were adjusted using the Benjamini–Hochberg (BH) method.

Temporal order of mutated gene

For a gene with recurrent nonsynonymous mutations (observed in at least two samples at both root and branch locations), we defined $p_0 = \frac{n_{root}}{N}$ and $p_1 = \frac{n_{branch}}{N}$, where n_{root} and n_{branch} are the numbers of mutations at the root and branch locations, respectively, and N is numbers of all mutations. We calculated the ratio p_1/p_0 to assess the likelihood of mutation timing.

Temporal pathway enrichment and association

In pathway enrichment analysis, we assigned each gene to Root, Branch or Incidental if at least 50% of its nonsynonymous mutations occurred at that location and retained only those genes for each location. The remaining genes were subjected to KEGG pathway enrichment analysis using clusterProfiler[13,14].

In pathway association analysis, we focused on nonsynonymous mutations in two locations—Root mutations and Branch mutations. Using curated KEGG pathway definitions, we constructed all possible pathway pairs. For each pair, we quantified the number of mutations occurring at Root and Branch. Pathways mutated in less than 1% of samples were excluded to focus the analysis on recurrently altered pathways. We then modeled the number of mutations in each pathway at t_1 as the response variable and those at t_0 as predictors. For pathway pairs that have shared genes, P-values were adjusted using the Benjamini–Hochberg (BH) method. For pathway pairs without shared genes, we relaxed the significance threshold by using unadjusted p-values and selected one pair as the representative. Temporal dependencies were interpreted based on the direction and magnitude of regression coefficients: positive slopes

indicated synergistic relationships, while negative slopes suggested antagonism. For clarity, we focused on synergistic relationships, defined as pathway pairs with positive regression coefficients.

Execution of programs

We downloaded the MOBSTER program from https://github.com/caravagnalab/MOBSTER. When applying MOBSTER to a tumor, the auto_setup parameters were set to FAST. We downloaded the TumE program from https://github.com/tomouellette/TumE. When applying TumE to a tumor, the parameters were set to default.

Approximation of tumor cell population size

At the time point t_e when a tumor is sampled for analysis, the total number of cells $N = \mathrm{N}_a + \mathrm{N}_f - N_l$ where N_a and N_f are the population sizes of K_a and K_f, respectively, and N_l is the number of cells that are counted twice when computing N_a and N_f and needs to be subtracted. By setting $t_0 = 0$, the tumor cell population size at t_e is

$$N = \mathrm{N}_a + \mathrm{N}_f - N_l = e^{\lambda\beta t_{end}} + e^{\lambda\beta(1+s)(t_e - t_f)} - e^{\lambda\beta(t_e - t_f)}$$

When the final tumor cell population size $N = \mathrm{N}_b + \mathrm{N}_f - N_l$ is approximated as $\widetilde{N} = \mathrm{N}_b + \mathrm{N}_f$, it leads to overestimation. Here we quantify the error as

$$error = \frac{N_l}{N} = \frac{1}{e^{\lambda\beta t_f} + e^{\lambda\beta s(t_e - t_f)} - 1}$$

Let $\lambda = \ln 2$ and $\beta = 1$. When $t_f > 5$ or $s(t_e - t_f) > 5$, the error is less than 1/31. Given that tumor cells undergo a large number of cell division cycles, it is reasonable to assume the error is negligible.

Initial decomposition of VAFs

Mutations are first split into two partitions using the MAGOS program[15]. Based on beta-distributions reparametrized with mean sequencing depth and mean VAF, MAGOS progressively groups mutations into a hierarchical tree structure. TEATIME traverses this tree,

calculating the mean VAF for each node. The first partition Ω_1 aggregates mutations in nodes with mean VAF>0.25, indicating presence in >50% of cells. The second partition Ω_2 contains the remaining mutations.

Within each partition, mutation are further split into clusters using the automixfit() function from the R/RBesT package[16]. The automixfit() function is configured with Nc=seq (1, 10) and k=6, modeling the VAFs as a mixture of beta distributions with 1 to 10 components. The shape parameters α and β for each identified component are returned. Using $v = \beta/(\alpha + \beta)$, each component is reparametrized as $\mathcal{B}(v)$. Mutations are then assigned to one of these components, which satisfies $\text{argmax}_{x \in V}\, prob(VAF|\mathcal{B}(v = x))$, where V is the collection of v values of the identified components. Mutations assigned to the same component form a cluster, with a mean VAF $\bar{V}$. It is noteworthy that while the $\bar{V}$ value of a cluster is close to the v value of the corresponding component, they are not necessarily equal. Furthermore, automixfit() requires the number of components is prespecified, which is unknown. Therefore, these clustering results provide only initial starting points for further optimization.

Joint optimization of mutation clusters and evolutionary parameters

Given the large number of unknowns, including mutation clusters and evolutionary parameters, an exhaustive search for optimal solutions is intractable. To address this problem, TEATIME carefully defines the search spaces for a subset of variables, iteratively selects possible values from these spaces, and uses them to estimate the remaining variables. Solutions from each iteration are compared, and the one showing the best fit between the observed VAFs and the inferred evolutionary process is selected as the final solution.

Specifically, if the branch cluster $U^b \sim \mathcal{B}(v^b)$ is located, P can be estimated as $2v^b$. Given the P value, v_t^k can be calculated for each $t \in [1,2, \dots, t_f - 1]$ and applying $f_k(t) = 2v_t^k$. Based on the series of v_t^k values, mutations can be grouped into a set of trunk clusters $U_t^k \sim \mathcal{B}(v_t^k)$. The

μ value can then be inferred either by counting mutations in the $U_{t=1}^{k}$ cluster or by estimating the slope of a line fitted across all U_t^k clusters based on Eq. (4).

We first define the search space for v^b. In a tumor dominated by K_f, i.e., $P > 0.5$, it is expected that $v^b > 0.25$, and the U^b cluster is in the Ω_1 partition. Among all clusters in Ω_1, Root cluster U^r cannot be U^b. To find U^r, the cluster with the highest mean VAF $\overline{V^h}$ is examined. If $\overline{V^h} \in [0.45, 0.55]$, this cluster is assigned as U^r. Otherwise, a new component $\mathcal{B}(v = 0.5)$ is created. Each VAF in Ω_1 is reassigned to one of the existing and new components, which satisfies $\operatorname{argmax}_{x\in\{\mathrm{V},0.5\}} prob(VAF|\mathcal{B}(v = x))$. The new cluster $U \sim \mathcal{B}(v = 0.5)$ is again split using the automixfit() function, and the cluster with the highest mean VAF is examined. This process continues till the $\overline{V^h}$ falls within the range $[0.45, 0.55]$. Upon completion, three clusters are recorded – U^r is the cluster with the highest mean VAF; U' is the cluster with the second highest mean VAF $\overline{V'}$; and U'' the one with lowest mean VAF $\overline{V''}$. Theoretically, v^b can be any value in the range $[\overline{V'}, \overline{V''}]$. Practically, to reduce computation load, only $\overline{V'}$, $\overline{V'}$, and $(\overline{V'} + \overline{V''})/2$ are considered.

In a tumor dominated by K_a, it is expected that $v^b < 0.25$, and U^b is the cluster with the highest mean VAF in the Ω_2 partition. Starting with the initial clusters in Ω_2, the one with the highest mean VAF is iteratively split using the automixfit() function until no new clusters can be produced. Upon complete, and the cluster with the highest mean VAF $\overline{V'''}$ is assigned as U^b.

The above steps produce four possible v^b values: $\overline{V'}$, $\overline{V'}$, $(\overline{V'} + \overline{V''})/2$, and $\overline{V'''}$, which constitute the search space for v^b. The search space for t_f includes all integers between 1 and the total number of mutations in Ω_1 that are outside the U^r cluster (i.e., non-root mutations).

For a given pair of v^b and t_f values selected from their respective search space, P and a series of v_t^k values, denoted as V^k, are estimated. Non-root mutations are grouped into clusters,

where the VAF of each mutation satisfies $\mathrm{argmax}_{x\in\{v^b,V^k\}}\, prob(VAF|\mathcal{B}(v=x))$. Based on the identified U^k clusters, two approaches are used to estimate μ.

The mutation counting approach relies on the $U_{t=1}^k$ cluster, because this cluster has the highest mean VAF among all U^k clusters, making it less likely to be mixed with incidental mutations and more likely to be exhaustively sequenced (**Supplementary Materials**). Given the $U_{t=1}^k$ cluster with a mean VAF $\overline{V_{t=1}^k}$, k observed VAFs closet to $\overline{V_{t=1}^k}$ are selected. Meanwhile, k simulated VAFs are randomly sampled from the $\mathcal{B}\left(v=\overline{V_{t=1}^k}\right)$ distribution. If the k observed VAFs come from trunk mutations acquired during the same cell division, their distribution should be similar to that of the k simulated VAFs. This hypothesis is tested using the Mann-Whitney test with a type-I error cutoff of 0.05 [17,18]. A series of k values are evaluated, ranging from 1 to the number of mutations in $U_{t=1}^k$. Those accepting the null hypothesis are retained as possible estimates for μ. If more than one k value is retained, they are ranked by the effect size measured by Cliff's delta score.

The line-fitting approach uses all U_t^k clusters. Based on Eq. (4), a line is fit between $-\ln\left(2\overline{V_t^k}-P\right)$ and $M_k(t)$, where $\overline{V_t^k}$ is the mean VAF of cluster U_t^k and $M_k(t)$ is cumulative counts of trunk mutations till time t. The mutation rate is calculated as $\mu=\lambda\beta\gamma$, where γ is the slope of the fitted line. Meanwhile, a set of simulated clusters are created by randomly sampling μ VAFs from each $\mathcal{B}\left(v=\overline{V_t^k}\right)$ distribution. A line is fit across these simulated clusters and the $\hat{\mu}$ is estimated from the slope using the same procedure described above. This simulated is repeated 1000 times, producing 1000 $\hat{\mu}$ values. One-group t test is used to compare the 1000 $\hat{\mu}$ values with the μ value estimated from the observed VAFs. If no significant difference is found, the μ value is retained.

The μ values estimated using the two approaches are compared. If consensus exists, the associated set of mutation clusters and evolutionary parameters are retained. If there is no

consensus, line-fitting results are retained. If one approach produces an empty set, results from the other approach are retained.

Each pair of v^b and t_f values produce estimates for mutation clusters and evolutionary parameters. After all pairs of values are tested, these estimates are compared on Bayesian Information Criterion scores $BIC = -2\log(\mathcal{L}) + k\ \log(m)$, where $\mathcal{L}$ is the likelihood that the observed VAFs belong to the set of $\mathcal{B}(v_t^k)$ distributions, k is the number of U^k clusters, and m is the number of mutations in U^k clusters. The set of estimates with the best BIC score is selected as the final solution.

Once P is known, s can be calculated using the mean VAF of cluster $U_{t_f+1}^s$, formed by successor mutations acquired at time $t_f + 1$, based on Eq. (5). In a tumor dominated by the K_f subclone, the $U_{t_f+1}^s$ cluster is expected to be in the Ω_2 partition. TEATIME searches for this cluster in the initial clusters identified in Ω_2. Given an arbitrary cluster in Ω_2 that has a mean VAF $< v^b$, it is assumed to be $U_{t_f+1}^s$, and s is calculated (**Supplementary Materials**). The number of $U_{t<t_f+1}^s$ clusters, each consisting of successor mutations acquired at a time point before $t_f + 1$, is given by $I = round(s)$. Based on the series of clusters U_i^s, where $i = 1, 2, \ldots, I$, all mutations between U^b and $U_{t_f+1}^s$ are regrouped to clusters. Using the updated $U_{t_f+1}^s$, a new selection coefficient value s' can be estimated. If $|s - s'| > 10^{-6}$, the s value is updated to s', and the steps of identifying U_t^s clusters and computing s' are repeated. The iterations end when $|s - s'| \leq 10^{-6}$ or 1,000 iterations are completed. Lastly, t_e is calculated using Eq. (3).

TEATIME does not estimate s, t_e for tumors dominated by the K_a clone because the $U_{t_f+1}^s$ cannot be located in the current framework.

Estimation of Evolutionary parameters in root, trunk and successive clusters

Assuming a tumor sample contains solely cancerous cells after adjusting for normal cell contamination, TEATIME models VAFs as a mixture of beta distributions. Each component in

the mixture is a beta distribution $\mathcal{B}(\alpha, \beta)$ with the shape parameters $\alpha = dv$ and $\beta = d(1 - v)$, where d is the mean sequencing depth and v is the mean VAF [15]. Since mutations in a tumor share similar, known sequencing depths, the beta distribution can be reparametrized as $\mathcal{B}(v)$. TEATIME decomposes the mixture to identify clusters of mutations that belong to different categories. We split VAF into two partitions: first partition Ω_1 aggregates mutations with VAF>0.25, indicating presence in >50% of cells. The second partition Ω_2 contains the remaining mutations. The observed VAF distribution in Ω_1 is modeled as a three component Beta-mixture:

$$p(VAF) = \pi_r \mathcal{B}(v^r) + \pi_t \sum_{t=1}^{t1-1} \mathcal{B}(v_t^k) + \pi_b \mathcal{B}(v^b)$$

subject to

$$\pi_r + \sum_{t=1}^{t1-1} \pi_t + \pi_b = 1$$

Where Root mutations form a cluster $U^r \sim \mathcal{B}(v^r)$. Trunk mutations, acquired at various time points, generate a series of clusters $U_t^k \sim \mathcal{B}(v_t^k)$,where $f_k(t) = 2v_t^k = P + (1 - P)\frac{1}{e^{\lambda\beta t}}$. Branch mutations, representing later evolutionary events, form a single cluster $U^b \sim \mathcal{B}(v^b)$. Here, $\{\pi_r, \pi_t, \pi_b\}$are the relative contributions and are inferred by maximum likelihood from the data. The fraction of K_f cells among all tumor cells is estimated as $f_h = 2v^b$.

The mutation rate is inferred from the trunk clusters using two approaches. The first approach quantifying the number of mutations in the earliest trunk cluster $U_{t=1}^k$. Assuming all mutations in this cluster have been sequenced, the number of variants gives an estimate of μ. The second approach aggregates information over multiple trunk clusters. Based on Eq. (4), a line can be fitted between $-\ln(f_k(t) - P)$ and $M_k(t)$ across the series of U^k clusters. The slope of this line is given by $\gamma = \mu/\lambda\beta$, where $\lambda = \ln 2$ and β is a user-specified cell survival rate

between 0 and 1. Consensus between two approaches is taken as the final estimates. Based on Eq. (4), $t_f = M_k/\mu$.

The selection coefficient s is inferred using successive mutations in Ω_2. Because the expected VAFs of successive mutations acquired at $t > t_f + 1$ are lower than $P/4$, and clusters formed by low-frequency mutations are difficult to identify, we limit the analysis to successive mutations acquired before or at $t_f + 1$. Compared to K_a cells that go through one generation of cell division in a unit time, the fitness advantage of K_f cells is reflected in the $MCRA_f$ cell and its descendants undergoing $1 + s$ generations of cell division between time points t_f and $t_f + 1$. Each generation produces a cluster, including the cluster $U^s_{t_{f+1}}$ that corresponds to mutations acquired at $t_f + 1$ and additional $I = round(s)$ clusters that correspond to mutations acquired in intermediate generations before $t_f + 1$. Based on Eq. (5), the selection coefficient can be estimated as

$$s = \frac{\ln\left(\frac{P}{2v^s_{t_f+1}}\right)}{\lambda\beta}$$

Subsequently, t_e can be calculated using Eq. (3).

The above estimations assume that the U^r, U^k, U^b, and U^s clusters have already been identified. However, in practice, locating these clusters is challenging. TEATIME employs a joint optimization algorithm that identifies informative clusters and estimates evolutionary parameters concurrently, maximizing the likelihood that the observed VAF distribution is generated by the inferred evolutionary process (**Supplementary Fig. 1, Methods and Materials**).

Mixing of incidental mutations in root and trunk clusters

If K_f is dominant, all mutations from the foundation lineage will reside in the Ω_1 partition. Some incidental mutations acquired by early progeny of the $MRCA_0$ cell and some successor mutations acquired by early progeny of the $MRCA_f$ cells may also fall into the Ω_1 partition due to the death of sister cells.

Here we quantify the extent of mixing of incidental mutations in the $U_{t=1}^{k}$ and U^{r}clusters. Let X represent the VAFs of incidental mutations generated by the i-th cell division in K_a, with the mean VAF as f_i. Let Y represents the VAFs of trunk mutations acquired at time $t = 1$, with the mean VAF as f_1. If cell proliferation rate is 2, f_1 is always higher than f_i , i.e., $\Delta_{\mathrm{i}} = f_i - f_1 < 0$, as these trunk mutations are present in all K_f cells and half of K_a cells. The probability that an incidental mutation acquired at i-th cell division mixes into $U_{t=1}^{k}$ and U^{r} is $prob(X > Y)$. To get a close-form approximation of this probability, a normal distribution approximation is employed[19] as

$$prob(X > Y) \approx \Phi\left(\frac{\Delta_i}{\sqrt{\mathrm{\sigma_x}^2 + \mathrm{\sigma_y}^2}}\right)$$

where Φ (.) is the cumulative density function of normal distribution $\mathcal{N}(0, 1)$. Given that the sequencing depth is typically significantly greater than 1, the variance of the beta distribution can be approximated as $\frac{v(1-v)}{d}$. Therefore,

$$\Phi\left(\frac{\Delta_i}{\sqrt{\sigma_x{}^2 + \sigma_y{}^2}}\right) = \Phi\left(\frac{\Delta_i \times \sqrt{d}}{\sqrt{2f_1(1 - \Delta_i - f_1) + \Delta_i - \Delta_i{}^2}}\right) \leq \Phi\left(\frac{\Delta_i \times \sqrt{d}}{\sqrt{\frac{1 - \Delta_i{}^2}{2}}}\right)$$

Although f_1 cannot assume the maximum value, the above estimate offers a way to establish an upper bound for the probability of mixing. The probabilities of VAFs of incidental mutations mixing into $U_{t=1}^{k}$ vary with the value of i and the proportion of the K_f subclone P.

We performed simulations to assess the mixing probability for a series of P values from 10% to 40% and sequencing depths from 30× to 1000×. We then estimated the $prob(X > Y)$ by taking random samples from the beta distribution (reflecting a realistic scenario) and from a normal distribution approximation (acting as a closed-form upper bound). As expected, the simulations show that the normal distribution approximation effectively provides an upper bound for estimating the level of mixing (**Supplementary Fig. 2**). When d=100 and survive rate = 1, it

is straightforward to see that when i is 1, the probability of mixing is 0.01595 if $P = 30\%$, and 0.23947 if $P = 10\%$. When i increases to 2, these probabilities change to 0.00027 for $P = 30\%$ and 0.00993 for $P = 10\%$. As i becomes sufficiently large, the probability of mixing decreases significantly, reaching as low as 5.3x10^{-9} for $P = 30\%$, and 0.00013 for $P = 10\%$.

Even if the proportion of K_f is sufficiently small, at most 0.25μ incidental mutations will be mixed into the $U_{t=1}^{k}$ cluster. However, this scenario is often impractical in actual cancer samples[20], suggesting that $U_{t=1}^{k}$ despite the potential for minor mixing, remains the most distinct and least contaminated cluster in the Ω_1 partition.

Validation of TEATIME inferences using paired primary-metastatic tumor:

We analyzed data from the Metastatic Breast Cancer Project [21], which performed whole-exome sequencing (WES) of paired primary and metastatic tumors. Among these samples, 17 primary tumors contained at least 50 somatic mutations. TEATIME successfully infer evolutionary parameters for 10 of them. We then found the corresponding metastatic tumors and applied the Pairtree program[22] to each pair to identify clusters of mutations and infer the seeding events underlying metastatic dissemination. In a primary-metastatic pair, mutations present in both tumors are acquired pre-seeding, while those unique to the metastatic tumor are acquired post-seeding.

When we examined the Pairtree results, we found many tumors might have experienced multiple seeding events. For example, in sample 1156, shared primary-metastatic mutations formed two clusters, implying two seeding events (Supplementary Fig. 11 A). In such cases, testing whether early dissemination is associated with more mutations acquired post-seeding would require unambiguous mapping of these mutations to individual seeding events, which is infeasible with bulk sequencing data from a single metastatic sample. Instead, we tested whether early dissemination is associated with fewer mutations acquired pre-seeding, consistent with a shorter pre-dissemination evolutionary period. Specifically, for each seeding

event inferred by Pairtree, we retrieved the corresponding pre-seeding mutations and their VAFs in the primary tumor. We mapped these mutations to the evolutionary events inferred by TEATIME with the most similar VAFs and classified them as early dissemination if occurring before the emergence of the derived subclone, or late dissemination otherwise. We compared the number of mutations between these two groups using a paired t-test. The result showed that early dissemination events indeed had significantly fewer pre-seeding mutations than late disseminations (P = 0.044, Supplementary Fig. 11B).

We also examined the five primary–metastatic pairs that exhibited a single seeding event. For each pair, we retrieved the VAFs of pre-seeding mutations in the primary tumor and mapped them to the evolutionary events inferred by TEATIME with the most similar VAF. Interestingly, four of these five pairs showed very early dissemination, with seeding events traced to near the formation of the ancestral clone, consistent with reports of early metastatic spread in previous studies[23–25]. Only one pair had the estimated seeding time close to the emergence of the derived subclone. We next retrieved post-seeding mutations in the metastatic tumors and normalized their counts by dividing over sample-specific sequencing depth and mutation rate to mitigate confounding effects. Using a one-sample t-test, we found that tumors with early dissemination had significantly more post-seeding mutations than tumors with late dissemination (P = 0.046, Supplementary Fig. 11C).

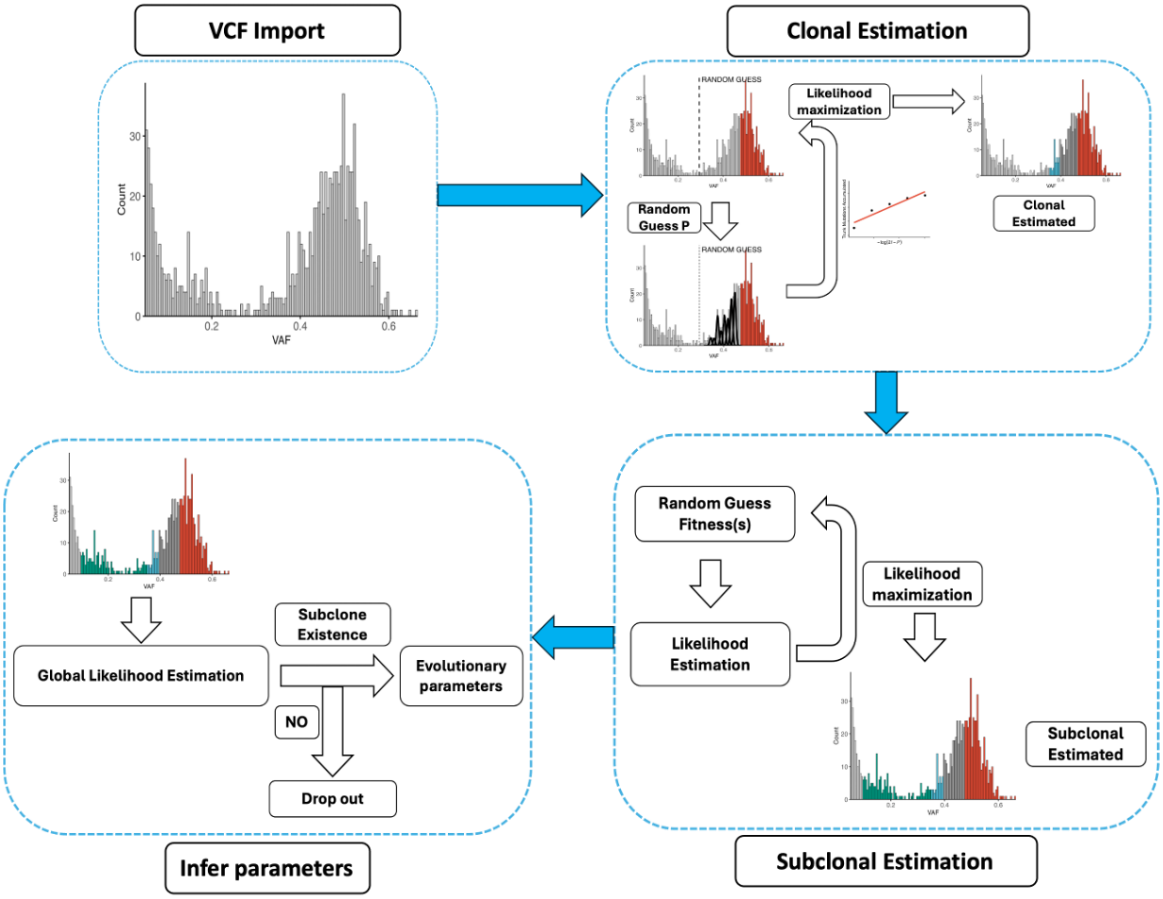

Supplementary Figure 1.
Flow chart describing the joint optimization process.

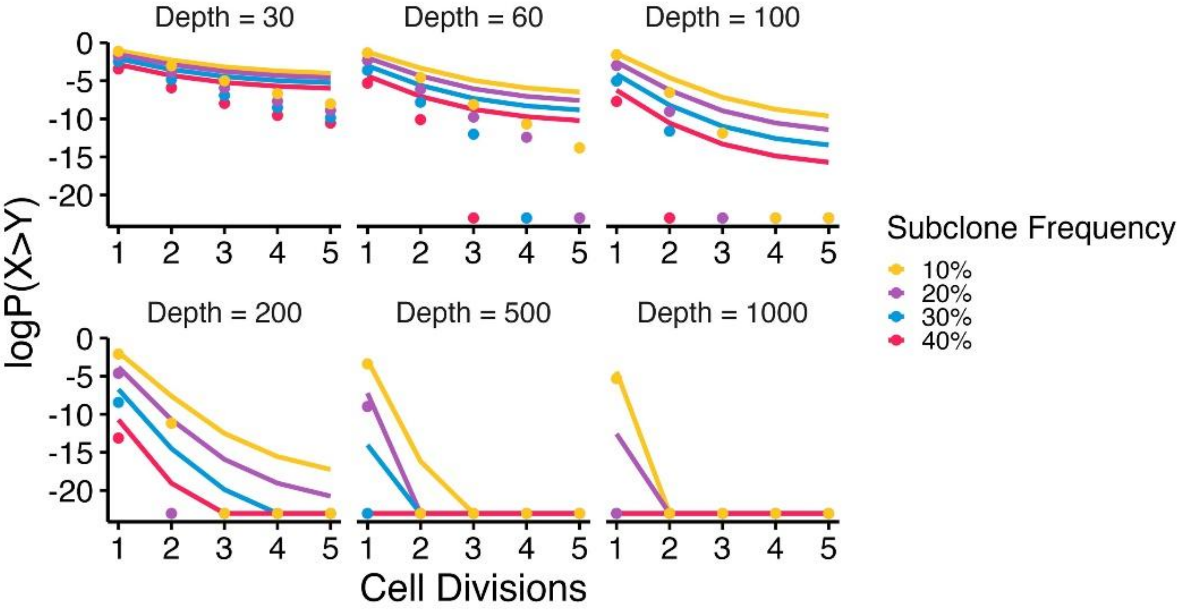

Supplementary Figure 2. Estimation of the probability of incidental mutations mixing into the root or first trunk clusters. The probability of mixing decreases with the cell division generations and sequencing depths. The line represents the normal distribution approximation, while the data points are derived from Beta distribution sampling. For clarity in visualization, probability $<10^{-10}$ is set to 10^{-10}.

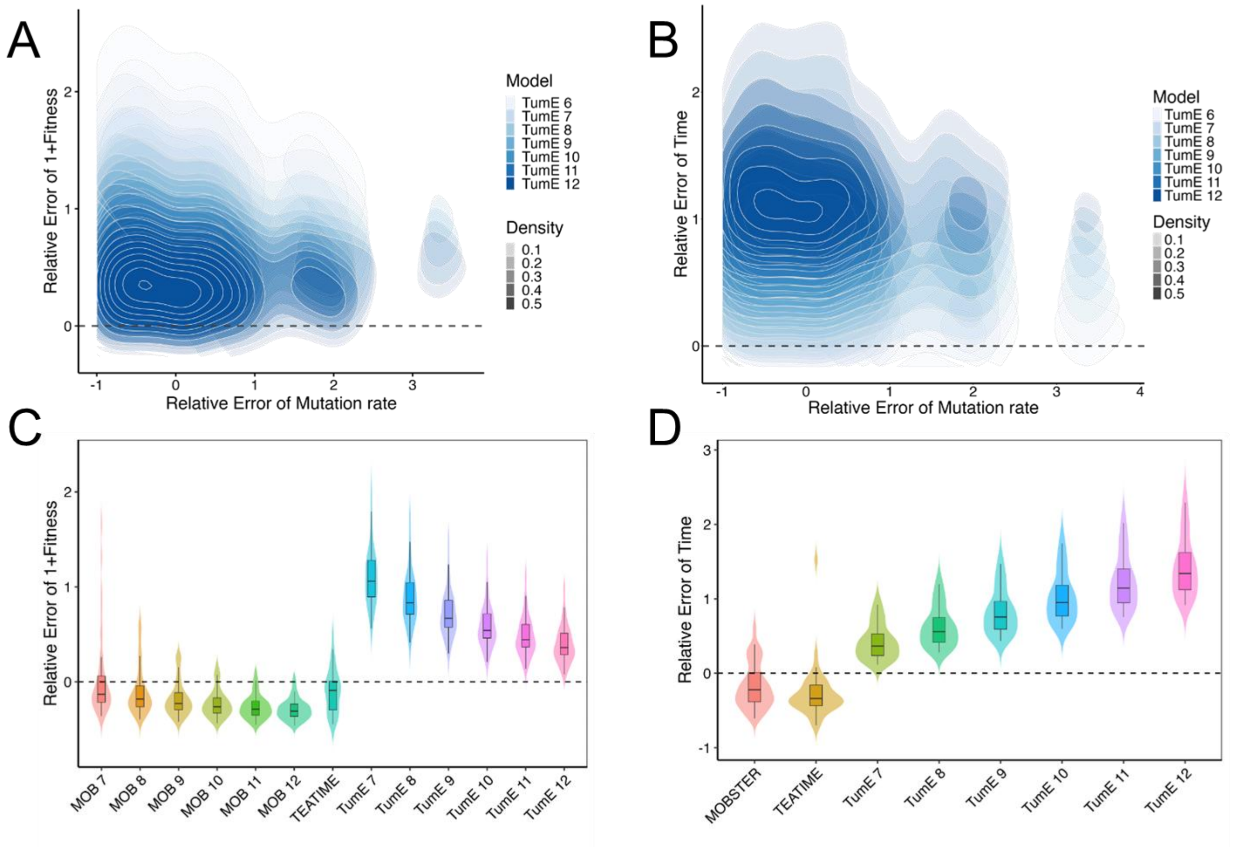

Supplementary Figure 3. Relative error of parameter estimates across different population sizes (N) for TumE, MOBSTER, and TEATIME.

(A-B) Relative errors of TumE Monte Carlo estimates for mutation rate (μ), fitness (s), and subclonal emergence time (t_1) across input population sizes ranging from 10^6 to 10^{12}. Colors represent different values of N.

(C-D) Comparison of relative errors in s, and t_1 estimates obtained from TumE (mean prediction), MOBSTER, and TEATIME. MOBSTER fitness prediction results at N = 10^6 were excluded due to instability and extreme error values.

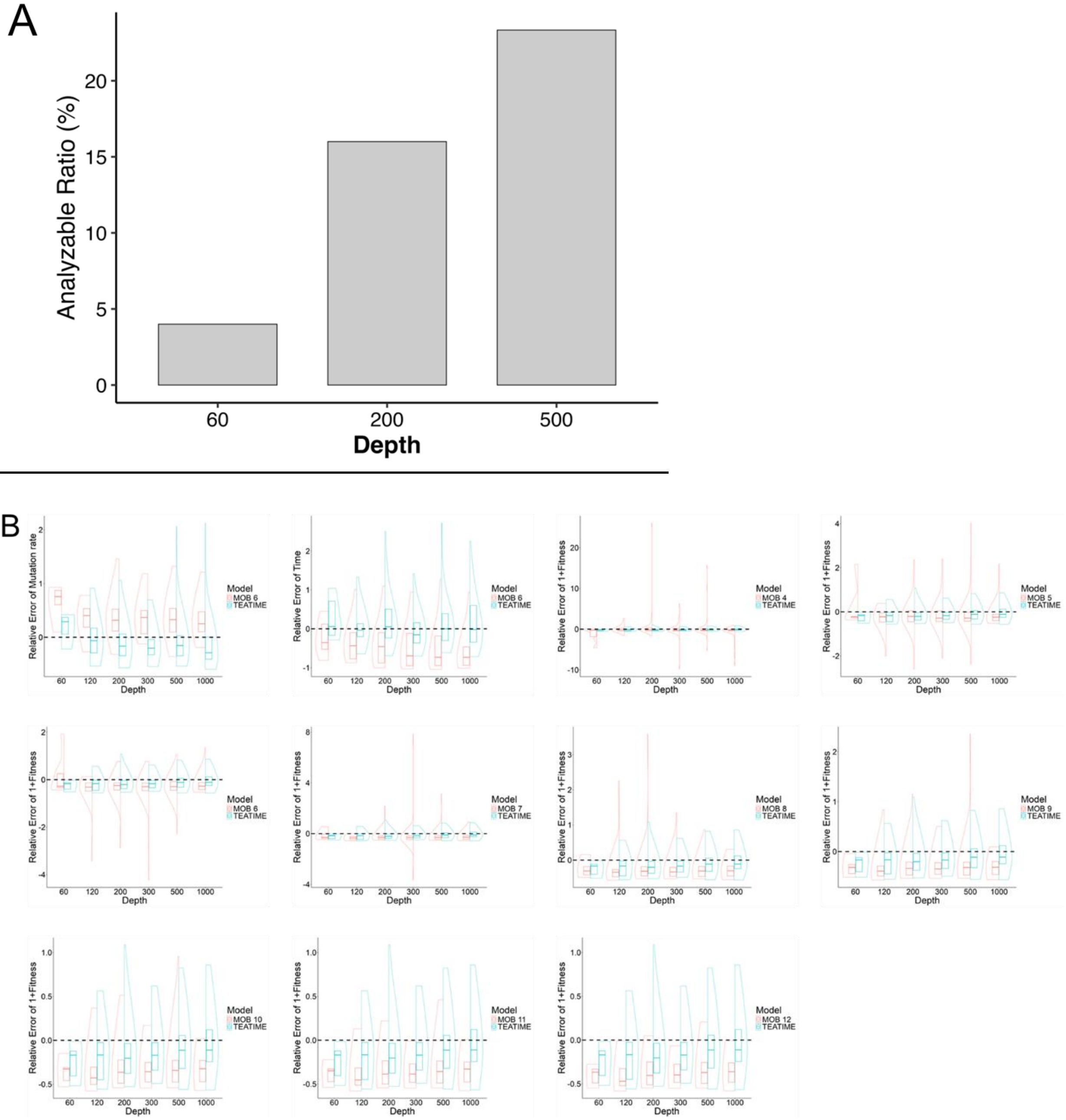
A
Analyzable Ratio (%)
0
5
10
15
20
60
200
500
Depth
B
Relative Error of Mutation rate
Relative Error of Time
Relative Error of 1+Fitness
Depth
60
120
200
300
500
1000
Model
MOB 4
MOB 5
MOB 6
MOB 7
MOB 8
MOB 9
MOB 10
MOB 11
MOB 12
TEATIME

C

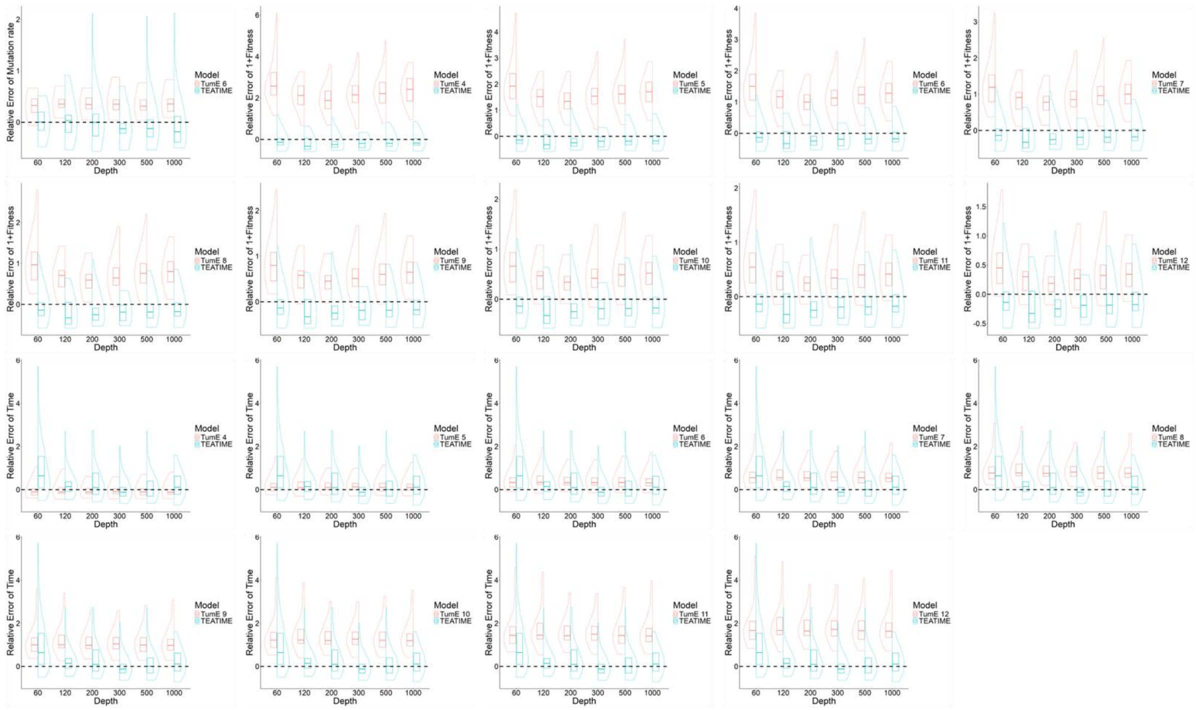

D

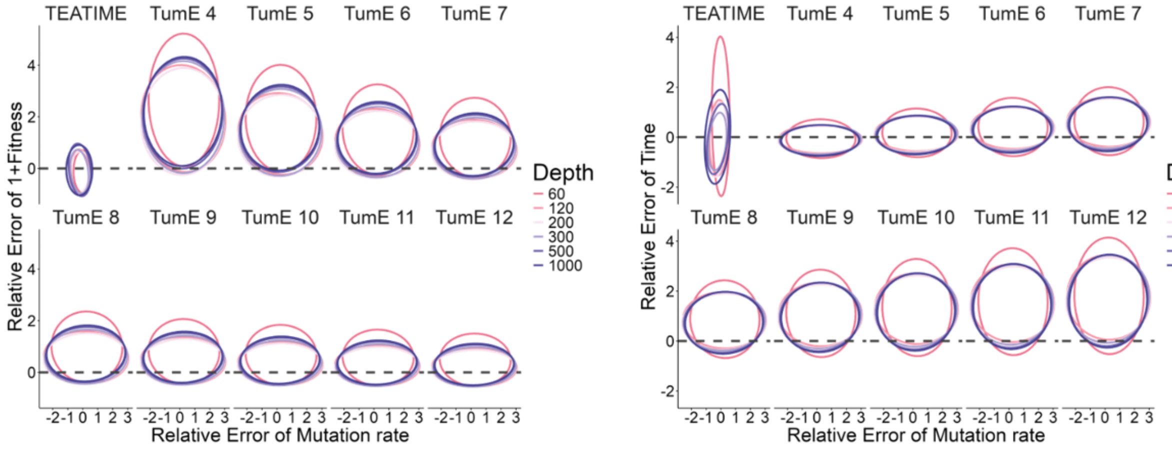

Supplementary Figure 4. Comparative analysis of the TEATIME method versus other models at population size N $\approx 10^6$ across different sequencing depths.

(A). Analyzable Ratio in different sequencing depths.

(B) Performance of MOBSTER using various N values, including 10^6, compared to TEATIME.

(C) Performance of TumE (mean predictions) using various N values, including 10^6, compared to TEATIME.

(D) 95% confidence ellipses showing the relative error distribution of parameter estimates

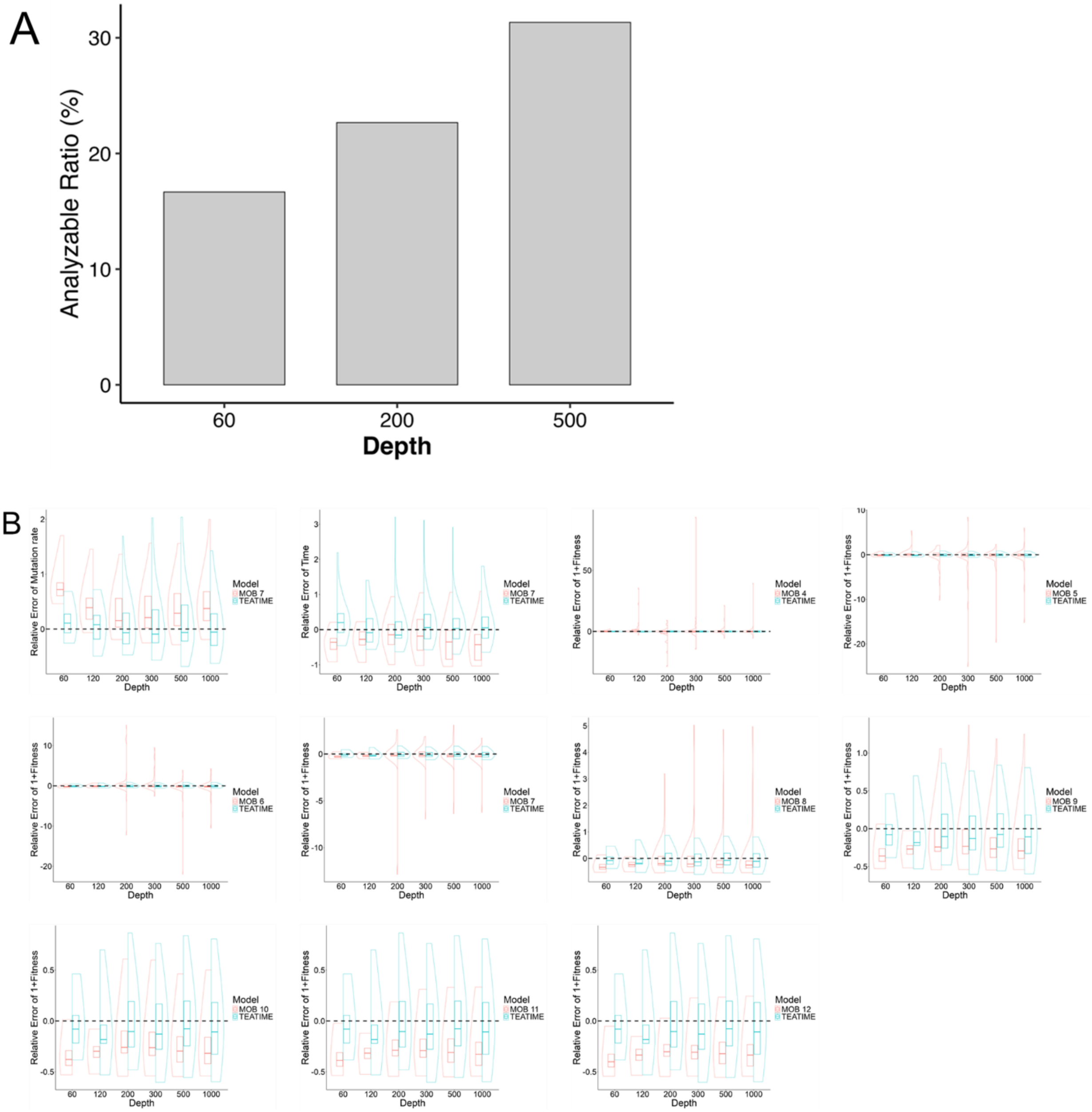
A
Analyzable Ratio (%)
0
10
20
30
60
200
500
Depth
B
Relative Error of Mutation rate
Relative Error of Time
Relative Error of 1+Fitness
Depth
60
120
200
300
500
1000
Model
MOB 4
MOB 5
MOB 6
MOB 7
MOB 8
MOB 9
MOB 10
MOB 11
MOB 12
TEATIME

C

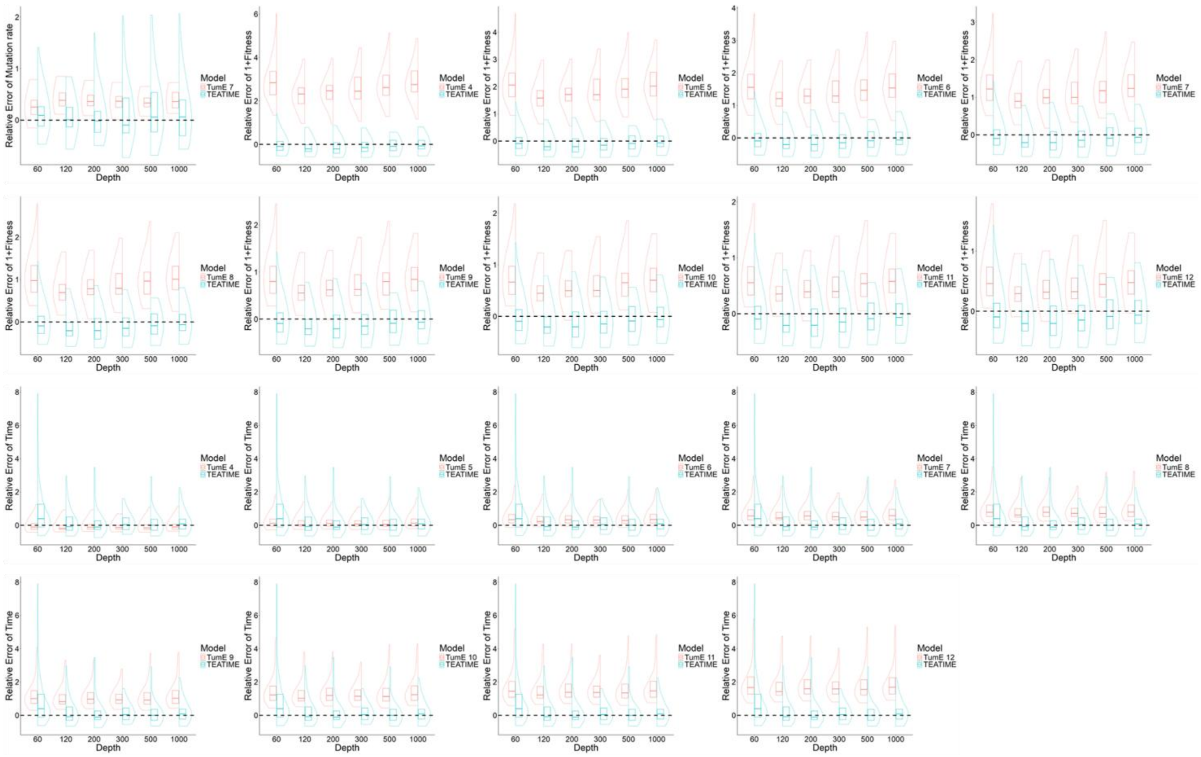

D

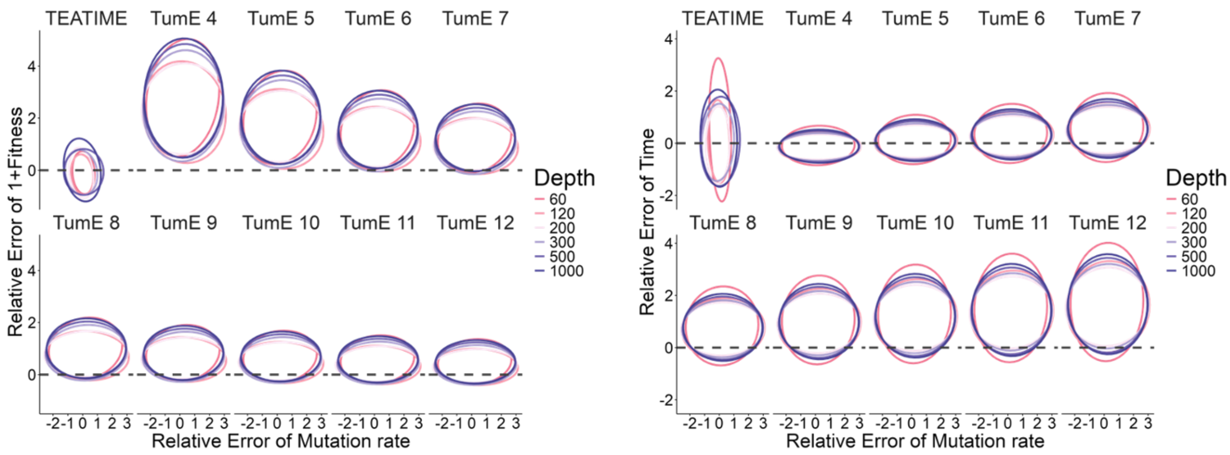

Supplementary Figure 5. Comparative analysis of the TEATIME method versus other models at population size N $\approx 10^7$ across different sequencing depths.

(A). Analyzable Ratio in different sequencing depths

(B) Performance of MOBSTER using various N values, including 10^7, compared to TEATIME.

(C) Performance of TumE (mean predictions) using various N values, including 10^7, compared to TEATIME.

(D) 95% confidence ellipses showing the relative error distribution of parameter estimates

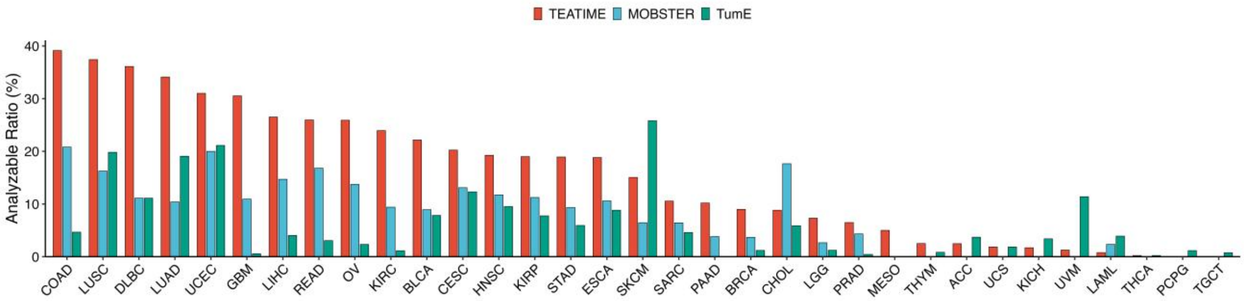

Supplementary Figure 6. Analyzable case ratio across tumor types in pan-cancer analysis. Tumor types are ordered by the proportion of predictable cases using TEATIME predictions.

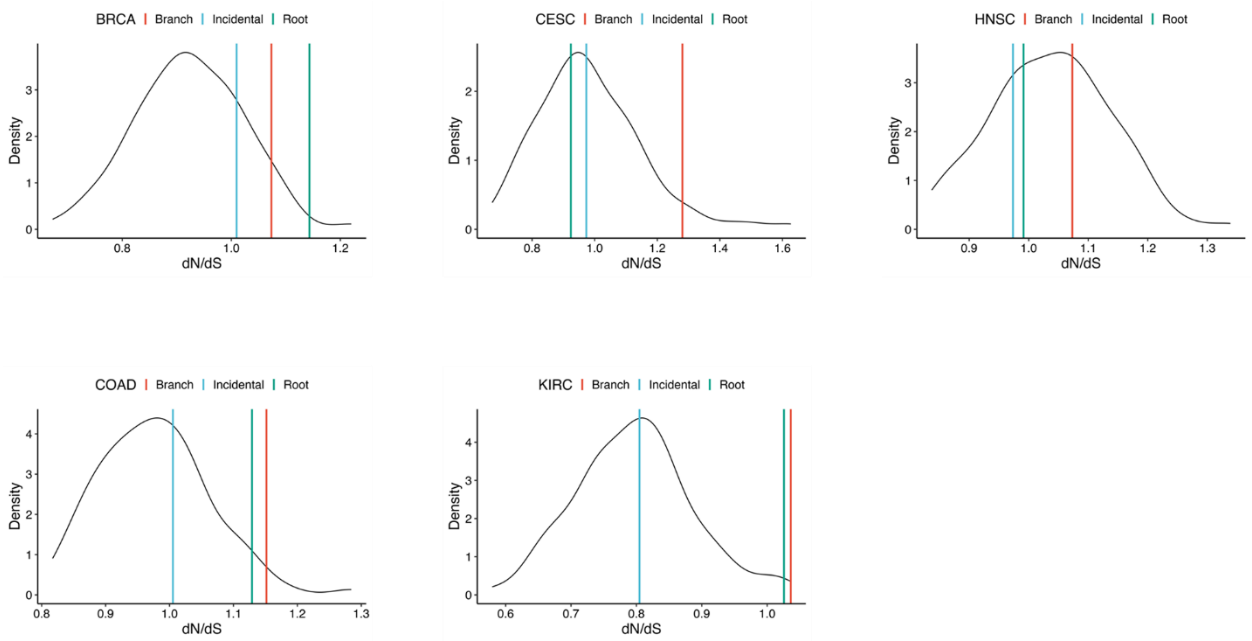

Supplementary Figure 7. dN/dS ratios in BRCA, COAD, CESC, HNSC, KIRC.

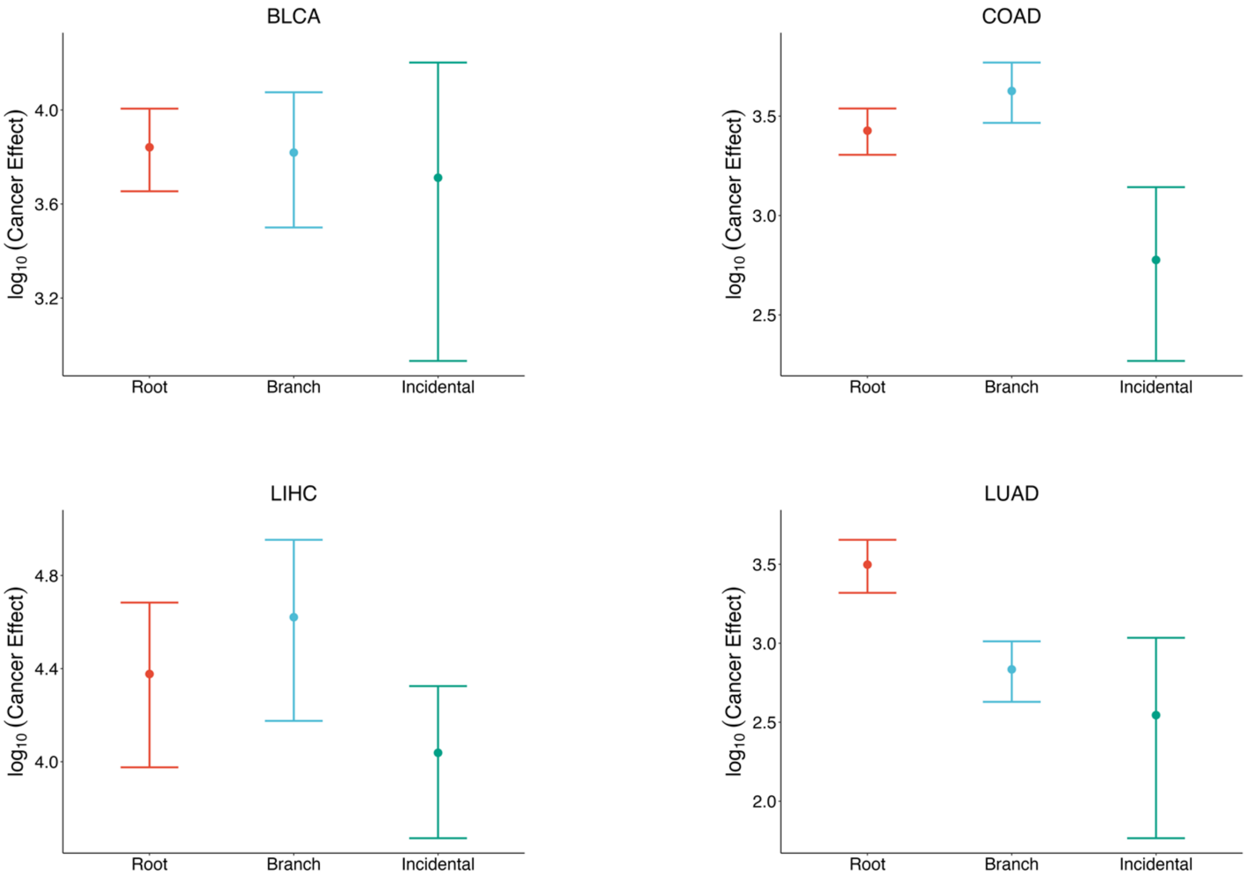

Supplementary Figure 8. Comparison of cancer effect size for Root, Branch and Incidental mutations across cancer types.

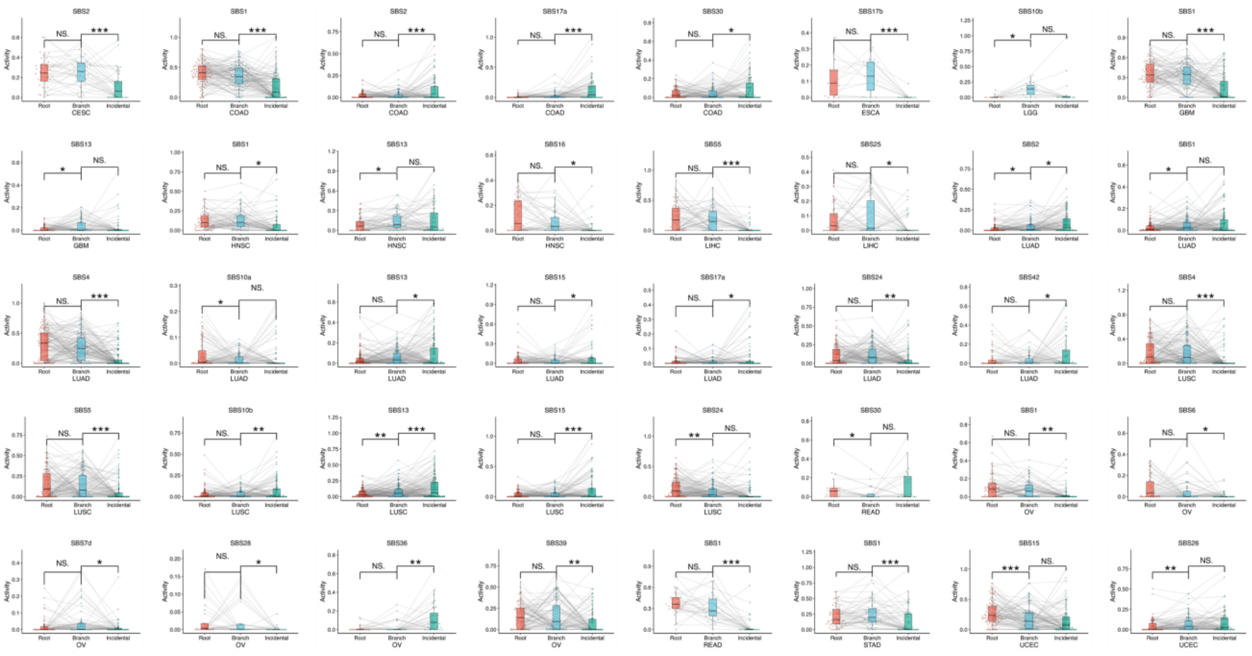

Supplementary Figure 9. Mutational signatures: Activity Changes moving from Root to Branch to Incidental mutations.

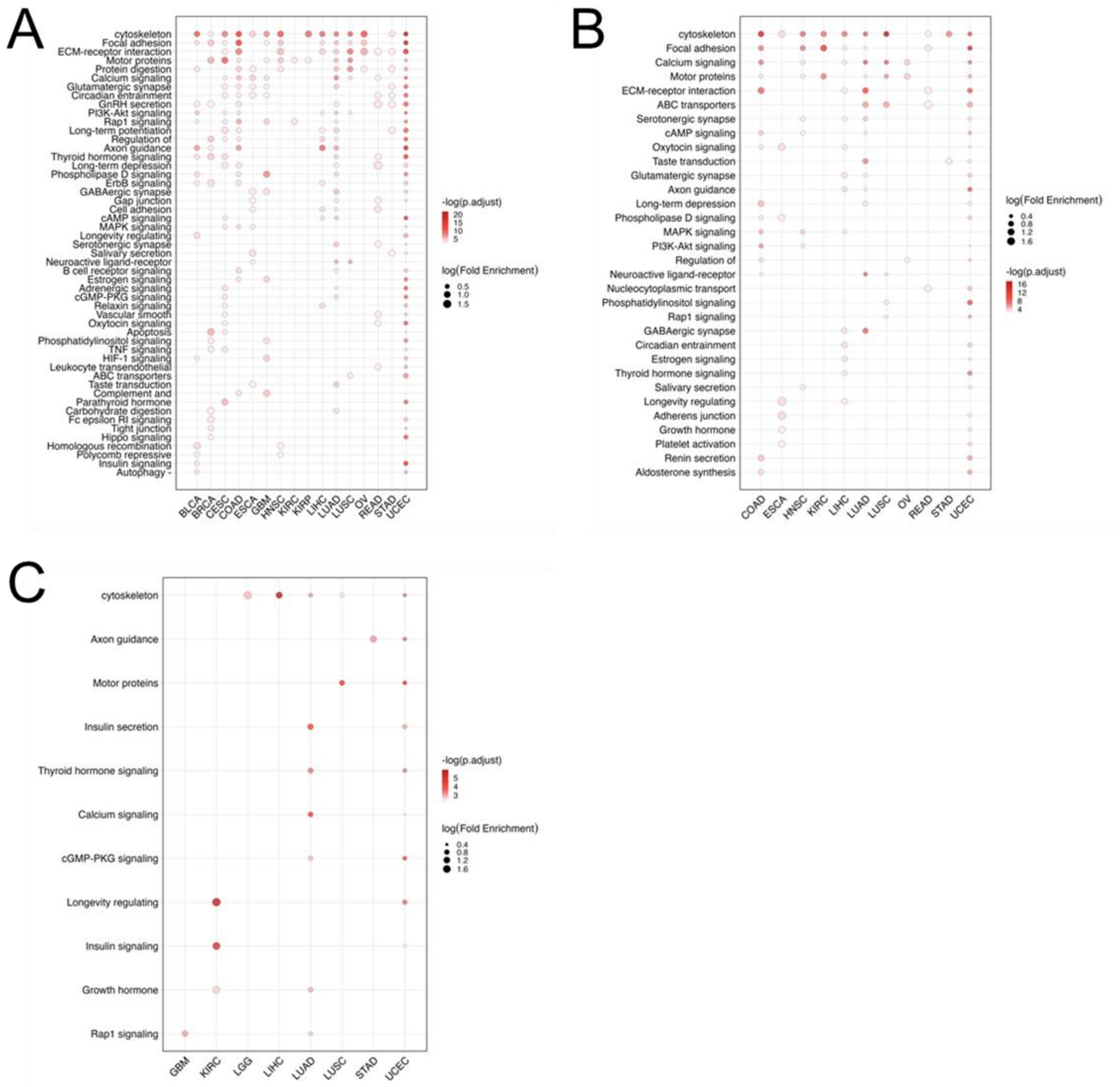

Supplementary Figure 10. Pan-cancer enrichment of pathways at Different Timepoint. (A) Enrichment on Root. (B) Enrichment on Branch. (C) Enrichment on Incidental. Only pathways significantly enriched in at least two cancer types are shown.

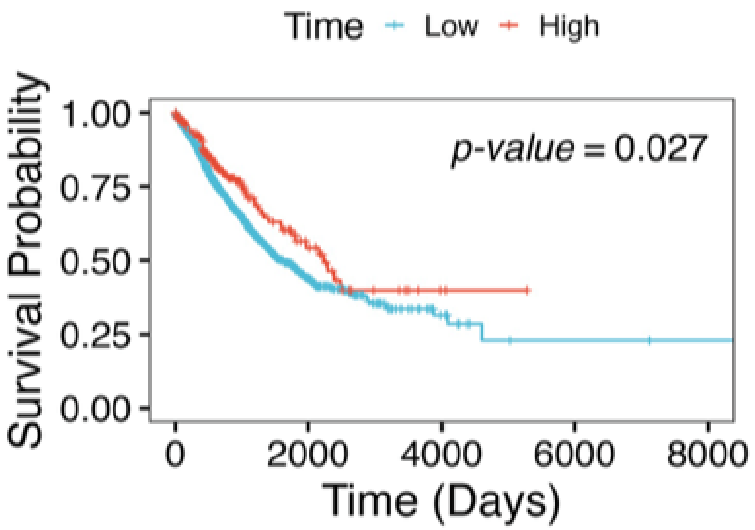

Supplementary Figure 11. Pan-cancer analysis assessing the association between time and overall survival. Low (early) vs High (late) groups.

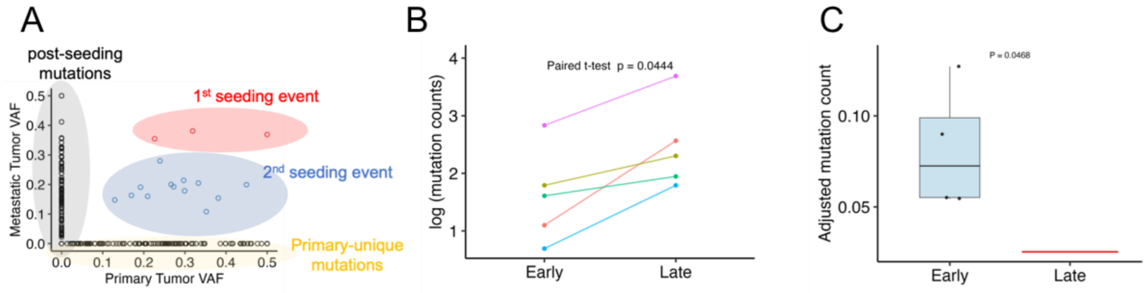

Supplementary Figure 12. Primary-Metastatic tumor comparison. (A) Primary-Metastatic VAF distribution. (B) Primary-Metastatic comparison on multiple seeding samples. (C) Primary-Metastatic comparison on single seeding samples.

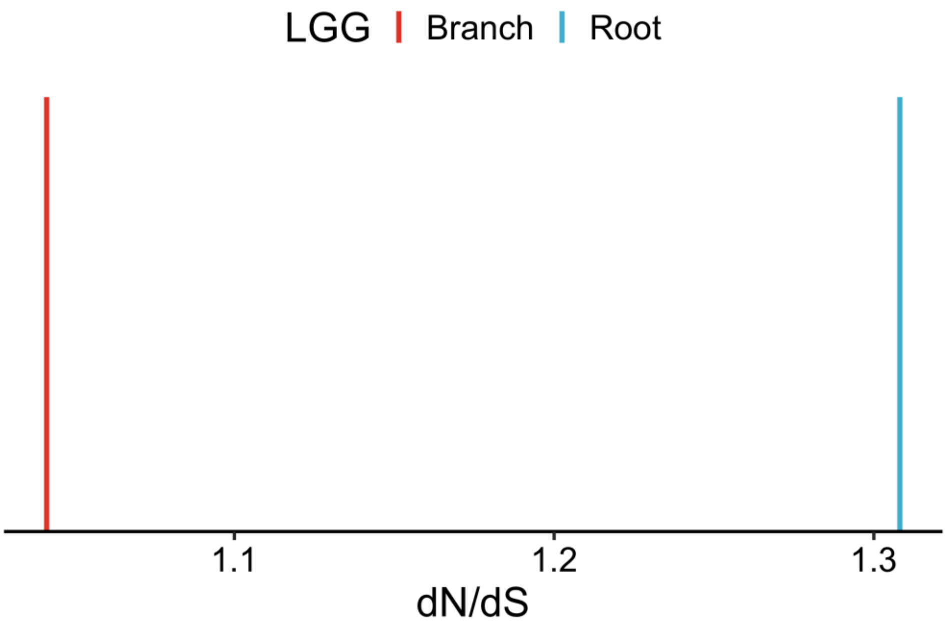

Supplementary Figure 13. LGG dN/dS in Root and Branch Mutations.

Supplementary Table 1. Primary tumor statistics.

		# of analyzable tumors		
Cancer Type	# of primary tumors	MOBSTER	TEATIME	TUME
ACC	81	0	2	3
BLCA	370	33	82	29
BRCA	923	34	83	11
CESC	252	33	51	31
CHOL	34	6	3	2
COAD	322	67	126	15
DLBC	36	4	13	4
ESCA	170	18	32	15
GBM	357	39	109	2
HNSC	452	53	87	43
KICH	59	0	1	2
KIRC	351	33	84	4
KIRP	258	29	49	20
LAML	128	3	1	5
LGG	492	13	36	6
LIHC	347	51	92	14
LUAD	519	54	177	99
LUSC	449	73	168	89
MESO	80	0	4	0
OV	386	53	100	9
PAAD	157	6	16	0
PCPG	173	0	0	2
PRAD	463	20	30	2
READ	131	22	34	4
SARC	218	14	23	10
SKCM	93	6	14	24
STAD	354	33	67	21
TGCT	131	0	0	1
THCA	451	0	1	1
THYM	120	0	3	1
UCEC	445	89	138	94
UCS	54	0	1	1
UVM	79	0	1	9

Supplementary Table 2. Mediation Test with immune infiltration

Immune infiltration	value_type	Mediator	acme_estimate	acme_p	ade_estimate	ade_p	total_estimate	total_p	prop_estimate	prop_p
B.cell_TIMER	binary	Fitness	-0.033976657	0.016	-0.16071075	0.018	-0.1946874	0.006	0.1760557	0.022
B.cell_TIMER	continuous	Fitness	-0.038598925	0.034	-0.16067056	0.028	-0.19926948	0.006	0.191354	0.04
B.cell_XCELL	binary	Fitness	-0.043565368	0	-0.27315125	0	-0.31671662	0	0.13774345	0
B.cell_XCELL	continuous	Fitness	-0.076189997	0	-0.26122126	0	-0.33741126	0	0.22459049	0
B.cell.memory_CIBERSORT.ABS	binary	Fitness	-0.042957523	0.022	-1.45005034	0	-1.49300787	0	0.02950463	0.022
B.cell.memory_CIBERSORT.ABS	continuous	Fitness	-0.198456926	0.032	-1.44255654	0	-1.64101347	0	0.11967468	0.032
B.cell.memory_XCELL	binary	Fitness	-0.044740534	0	-0.79817391	0	-0.84291444	0	0.05338261	0
B.cell.memory_XCELL	continuous	Fitness	-0.190042052	0	-0.78569872	0	-0.97574077	0	0.19335793	0
B.cell.naive_XCELL	binary	Fitness	-0.045545378	0.002	-1.54153938	0.012	-1.58708476	0.012	0.02891556	0.014
B.cell.naive_XCELL	continuous	Fitness	-0.429614341	0.01	-1.56310343	0.01	-1.99271777	0	0.21578302	0.01
Cancer.associated.fibroblast_XCELL	binary	Fitness	-0.034525875	0.012	-0.40837481	0	-0.44290068	0	0.07993316	0.012
Cancer.associated.fibroblast_XCELL	continuous	Fitness	-0.061493954	0.012	-0.4176469	0	-0.47914085	0	0.12750787	0.012
Hematopoietic.stem.cell_XCELL	continuous	Fitness	-0.03258979	0.032	-0.08460473	0.086	-0.11719452	0.02	0.27326683	0.052
immune.score_XCELL	binary	Fitness	-0.040932135	0	-0.17167274	0	-0.21260487	0	0.19128675	0
immune.score_XCELL	continuous	Fitness	-0.068229166	0	-0.15656165	0.002	-0.22479082	0	0.30219096	0
Macrophage_TIMER	continuous	Fitness	-0.057030504	0.006	-0.10860026	0.146	-0.16563076	0.034	0.33211836	0.04
Macrophage_XCELL	binary	Fitness	-0.046650426	0.004	-0.26789655	0.062	-0.31454698	0.024	0.14811058	0.028
Macrophage_XCELL	continuous	Fitness	-0.186409905	0	-0.20610143	0.118	-0.39251133	0.006	0.48290983	0.006
Macrophage_XCELL	binary	Time	-0.010715692	0.03	-0.37686664	0	-0.38758233	0	0.02665683	0.03
Macrophage.M1_CIBERSORT.ABS	binary	Fitness	-0.04690493	0	-0.44772801	0.002	-0.49463294	0	0.09527794	0
Macrophage.M1_CIBERSORT.ABS	continuous	Fitness	-0.173655147	0	-0.38686175	0.022	-0.5605169	0	0.30672657	0
Macrophage.M1_XCELL	binary	Fitness	-0.047461763	0.002	-0.58554799	0.002	-0.63300975	0.002	0.07603096	0.004
Macrophage.M1_XCELL	continuous	Fitness	-0.283491016	0	-0.49876685	0.018	-0.78225786	0	0.35740113	0
Macrophage.M1_XCELL	binary	Time	-0.012142612	0.026	-0.73317137	0	-0.74531398	0	0.01617303	0.026
Macrophage.M2_CIBERSORT.ABS	continuous	Fitness	-0.067477249	0	-0.06717743	0.298	-0.13465468	0.042	0.48453298	0.042
Macrophage.M2_XCELL	binary	Fitness	-0.046808203	0.018	-0.50755653	0.038	-0.55436474	0.028	0.08347552	0.046
Macrophage.M2_XCELL	continuous	Fitness	-0.233219114	0.002	-0.42296778	0.098	-0.65618689	0.006	0.35398861	0.008
Macrophage.M2_XCELL	binary	Time	-0.01376677	0.026	-0.56277729	0.026	-0.57654405	0.022	0.02353836	0.048
microenvironment.score_XCELL	binary	Fitness	-0.041320321	0	-0.18565454	0	-0.22697486	0	0.18029796	0
microenvironment.score_XCELL	continuous	Fitness	-0.065459483	0	-0.17224883	0	-0.23770832	0	0.27810751	0
microenvironment.score_XCELL	binary	Time	-0.007558952	0.044	-0.23430585	0	-0.24186481	0	0.03065497	0.044
Monocyte_XCELL	binary	Fitness	-0.045390058	0.002	-0.44329524	0.004	-0.4886853	0	0.09236373	0.002
Monocyte_XCELL	continuous	Fitness	-0.19919794	0	-0.38740315	0.016	-0.58660109	0	0.33444572	0
Monocyte_XCELL	binary	Time	-0.010965257	0.036	-0.58325067	0	-0.59421593	0	0.0183946	0.036
Myeloid.dendritic.cell_TIMER	binary	Fitness	-0.024528784	0	-0.08027054	0.004	-0.10479933	0	0.23423425	0
Myeloid.dendritic.cell_TIMER	continuous	Fitness	-0.036898163	0	-0.06940229	0.006	-0.10630045	0	0.3488855	0
Myeloid.dendritic.cell_TIMER	binary	Time	-0.003826345	0.022	-0.09651426	0.002	-0.1003406	0.002	0.03574446	0.024
Myeloid.dendritic.cell_XCELL	binary	Fitness	-0.04249724	0.038	-0.51690563	0.008	-0.55940287	0.002	0.07811728	0.04
Myeloid.dendritic.cell_XCELL	continuous	Fitness	-0.167072359	0.012	-0.46880502	0.024	-0.63587738	0.004	0.26226418	0.016
Myeloid.dendritic.cell.activated_XCEL	binary	Fitness	-0.039518015	0	-0.16332781	0.002	-0.20284582	0	0.19473056	0
Myeloid.dendritic.cell.activated_XCEL	continuous	Fitness	-0.060291624	0	-0.14748131	0	-0.20777293	0	0.28993353	0
Neutrophil_TIMER	binary	Fitness	-0.049379275	0	-0.24454441	0.002	-0.29392368	0	0.16983177	0
Neutrophil_TIMER	continuous	Fitness	-0.114074208	0	-0.20908076	0.01	-0.32315497	0	0.35013614	0
Neutrophil_TIMER	binary	Time	-0.008747558	0.03	-0.30810004	0	-0.3168476	0	0.02624185	0.03
Plasmacytoid.dendritic.cell_XCELL	binary	Fitness	-0.047480882	0	-0.30448238	0.042	-0.35196326	0.018	0.13613797	0.018
Plasmacytoid.dendritic.cell_XCELL	continuous	Fitness	-0.19338888	0	-0.27288356	0.046	-0.46627244	0	0.41699518	0
stroma.score_XCELL	binary	Fitness	-0.040050483	0.014	-0.25404321	0.008	-0.2940937	0	0.13890811	0.014
stroma.score_XCELL	continuous	Fitness	-0.080577364	0.01	-0.24383436	0.014	-0.32441173	0	0.24755821	0.01
stroma.score_XCELL	binary	Time	-0.013678173	0.012	-0.29198337	0	-0.30566154	0	0.04467335	0.012
T.cell.CD4._TIMER	continuous	Fitness	-0.038846557	0.024	-0.11406221	0.066	-0.15290876	0.014	0.25501937	0.038
T.cell.CD4._TIMER	binary	Time	-0.00836564	0.04	-0.13346134	0.042	-0.14182697	0.028	0.05572147	0.064
T.cell.CD4..naive_XCELL	binary	Fitness	-0.044653797	0.006	-1.07095515	0.004	-1.11560894	0.002	0.03995882	0.008
T.cell.CD4..naive_XCELL	continuous	Fitness	-0.245364372	0.012	-1.0660363	0	-1.31140067	0	0.18686905	0.012
T.cell.CD4..Th2_XCELL	binary	Fitness	-0.024597249	0.026	-0.07284338	0.078	-0.09744063	0.024	0.24270039	0.05
T.cell.CD4..Th2_XCELL	continuous	Fitness	-0.050183825	0	-0.05145613	0.2	-0.10163996	0.016	0.48862705	0.016
T.cell.CD8._CIBERSORT.ABS	binary	Fitness	-0.047861608	0	-0.14831307	0.104	-0.19617468	0.034	0.2399073	0.034
T.cell.CD8._CIBERSORT.ABS	continuous	Fitness	-0.096059117	0	-0.12489569	0.168	-0.22095481	0.028	0.42349322	0.028
T.cell.CD8._CIBERSORT.ABS	binary	Time	-0.009111001	0.036	-0.2318176	0.006	-0.2409286	0.004	0.03660359	0.04
T.cell.CD8._TIMER	binary	Fitness	-0.040181893	0	-0.06160205	0.102	-0.10178395	0.012	0.40171411	0.012
T.cell.CD8._TIMER	continuous	Fitness	-0.0563643	0	-0.05537299	0.176	-0.1117373	0.008	0.50843941	0.008
T.cell.CD8._TIMER	binary	Time	-0.006383363	0.028	-0.09092982	0.024	-0.09731319	0.014	0.06266387	0.038
T.cell.CD8._XCELL	binary	Time	-0.009334572	0.04	-0.25476425	0.042	-0.26409882	0.034	0.03328804	0.066
T.cell.CD8..central.memory_XCELL	binary	Fitness	-0.045557383	0	-0.18559481	0.038	-0.2311522	0.008	0.19499893	0.008
T.cell.CD8..central.memory_XCELL	continuous	Fitness	-0.096962242	0	-0.17087923	0.044	-0.26784147	0	0.36711751	0

Supplementary Table 3. Multivariable regression with immune infiltration

method	Predictor	Immune infiltration	tumor	Standardized Estimate	pval_adj
TIMER	Diversity	B.cell_TIMER	SARC	-0.5434479	0.0500877
TIMER	Fitness	B.cell_TIMER	HNSC	-0.308881	0.07253466
TIMER	Fitness	B.cell_TIMER	LUSC	-0.2020764	0.04725699
XCELL	Fitness	B.cell_XCELL	LUSC	-3.62E-01	0.00027253
CIBERSORT	Diversity	B.cell.memory_CIBERSORT	LGG	-5.78E-01	0.01483937
CIBERSORT	Time	B.cell.memory_CIBERSORT	LGG	6.67E-01	0.00178503
CIBERSORT	Diversity	B.cell.memory_CIBERSORT.ABS	LGG	-4.87E-01	0.07990545
CIBERSORT	Time	B.cell.memory_CIBERSORT.ABS	LGG	5.60E-01	0.02322822
XCELL	Fitness	B.cell.memory_XCELL	LUSC	-3.30E-01	0.00090717
CIBERSORT	Diversity	B.cell.naive_CIBERSORT.ABS	SARC	-5.77E-01	0.08517683
XCELL	Time	B.cell.naive_XCELL	LUSC	1.88E-01	0.09805491
XCELL	Fitness	B.cell.plasma_XCELL	LUSC	-3.24E-01	0.00090717
XCELL	Fitness	Cancer.associated.fibroblast_XCELL	LUSC	-2.17E-01	0.05030834
XCELL	Diversity	Class.switched.memory.B.cell_XCELL	SARC	-5.67E-01	0.07940192
XCELL	Fitness	Class.switched.memory.B.cell_XCELL	LUSC	-3.01E-01	0.00216981
XCELL	Diversity	Common.lymphoid.progenitor_XCELL	LUSC	-1.94E-01	0.08644337
XCELL	Fitness	Common.lymphoid.progenitor_XCELL	UCEC	-2.57E-01	0.08058452
XCELL	Time	Endothelial.cell_XCELL	LUSC	2.17E-01	0.05030834
XCELL	Fitness	Eosinophil_XCELL	UCEC	2.56E-01	0.08058452
XCELL	Diversity	Granulocyte.monocyte.progenitor_XCELL	BRCA	-3.51E-01	0.0349398
XCELL	Time	Granulocyte.monocyte.progenitor_XCELL	LUSC	2.63E-01	0.00980389
XCELL	Fitness	Hematopoietic.stem.cell_XCELL	LGG	-6.17E-01	0.03714285
XCELL	Fitness	immune.score_XCELL	LUSC	-3.14E-01	0.00118415
TIMER	Fitness	Macrophage_TIMER	BLCA	-0.2152666	0.0997433
TIMER	Fitness	Macrophage_TIMER	LGG	-0.4600435	0.03511876
TIMER	Time	Macrophage_TIMER	BLCA	0.2643012	0.05268092
CIBERSORT	Time	Macrophage.M1_CIBERSORT	UCEC	2.73E-01	0.05422294
CIBERSORT	Fitness	Macrophage.M1_CIBERSORT.ABS	LUSC	-3.05E-01	0.01169148
XCELL	Fitness	Macrophage.M1_XCELL	LUSC	-2.36E-01	0.02912376
XCELL	Fitness	Macrophage.M2_XCELL	LGG	-5.82E-01	0.02403316
CIBERSORT	Diversity	Mast.cell.activated_CIBERSORT.ABS	SARC	-6.47E-01	0.08517683
XCELL	Fitness	microenvironment.score_XCELL	LUSC	-3.27E-01	0.00090717
XCELL	Fitness	Monocyte_XCELL	LUSC	-1.97E-01	0.07693762
TIMER	Diversity	Myeloid.dendritic.cell_TIMER	BLCA	-0.268178	0.06304311
TIMER	Fitness	Myeloid.dendritic.cell_TIMER	BLCA	-0.2299594	0.0986931
TIMER	Fitness	Myeloid.dendritic.cell_TIMER	HNSC	-0.3627937	0.03311552
TIMER	Fitness	Myeloid.dendritic.cell_TIMER	KIRC	-0.2612224	0.0915597
TIMER	Fitness	Myeloid.dendritic.cell_TIMER	LUSC	-0.2321259	0.02515552
TIMER	Time	Myeloid.dendritic.cell_TIMER	BLCA	0.3460602	0.0213897
TIMER	Time	Myeloid.dendritic.cell_TIMER	KIRC	0.2502129	0.0915597
XCELL	Diversity	Myeloid.dendritic.cell_XCELL	SARC	-6.43E-01	0.07940192
XCELL	Fitness	Myeloid.dendritic.cell.activated_XCELL	LUSC	-2.87E-01	0.00369495
CIBERSORT	Time	Myeloid.dendritic.cell.resting_CIBERSORT	LIHC	4.44E-01	0.00274512
CIBERSORT	Diversity	Myeloid.dendritic.cell.resting_CIBERSORT	KIRP	-4.82E-01	0.08799655
CIBERSORT	Time	Myeloid.dendritic.cell.resting_CIBERSORT	LIHC	5.01E-01	0.00032631
CIBERSORT	Diversity	Neutrophil_CIBERSORT	KIRP	-4.61E-01	0.08799655
CIBERSORT	Diversity	Neutrophil_CIBERSORT.ABS	KIRP	-0.4547739	0.08799655
TIMER	Diversity	Neutrophil_TIMER	BLCA	-0.2568405	0.06304311
TIMER	Fitness	Neutrophil_TIMER	BLCA	-0.2871608	0.05268092
TIMER	Fitness	Neutrophil_TIMER	KIRC	-0.302086	0.0915597
TIMER	Fitness	Neutrophil_TIMER	LUSC	-0.2916298	0.00350915
TIMER	Time	Neutrophil_TIMER	BLCA	0.2614325	0.05268092
TIMER	Time	Neutrophil_TIMER	KIRC	0.2469408	0.0915597
CIBERSORT	Diversity	NK.cell.resting_CIBERSORT	LGG	-4.61E-01	0.09965729
CIBERSORT	Diversity	NK.cell.resting_CIBERSORT.ABS	LGG	-4.87E-01	0.07990545
XCELL	Fitness	Plasmacytoid.dendritic.cell_XCELL	LUSC	-2.39E-01	0.02673625
XCELL	Fitness	stroma.score_XCELL	LUSC	-2.14E-01	0.05030834
XCELL	Time	stroma.score_XCELL	LUSC	2.16E-01	0.05030834
TIMER	Diversity	T.cell.CD4._TIMER	BRCA	-0.289421	0.08199004
TIMER	Diversity	T.cell.CD4._TIMER	SARC	-0.7550584	0.00683908
TIMER	Fitness	T.cell.CD4._TIMER	BLCA	-0.2786026	0.05268092
TIMER	Fitness	T.cell.CD4._TIMER	LUSC	-0.2252206	0.02515552
XCELL	Diversity	T.cell.CD4...non.regulatory._XCELL	BRCA	-3.99E-01	0.0349398
XCELL	Time	T.cell.CD4..central.memory_XCELL	BRCA	3.35E-01	0.06019715
XCELL	Diversity	T.cell.CD4..effector.memory_XCELL	SARC	-6.12E-01	0.07940192
XCELL	Fitness	T.cell.CD4..effector.memory_XCELL	LUSC	-2.36E-01	0.02673625
XCELL	Time	T.cell.CD4..effector.memory_XCELL	UCEC	2.55E-01	0.08058452
XCELL	Diversity	T.cell.CD4..memory_XCELL	SARC	-5.50E-01	0.07940192
CIBERSORT	Diversity	T.cell.CD4..memory.resting_CIBERSORT.A	SARC	-0.727183	0.0264224
XCELL	Diversity	T.cell.CD4..naive_XCELL	BRCA	-3.31E-01	0.06019715
XCELL	Diversity	T.cell.CD4..naive_XCELL	SARC	-5.56E-01	0.07940192
XCELL	Fitness	T.cell.CD4..naive_XCELL	LUSC	-2.05E-01	0.06181299
XCELL	Time	T.cell.CD4..naive_XCELL	UCEC	2.68E-01	0.08058452
TIMER	Diversity	T.cell.CD8._TIMER	BRCA	-3.13E-01	0.08199004
TIMER	Diversity	T.cell.CD8._TIMER	SARC	-5.20E-01	0.0500877
TIMER	Time	T.cell.CD8._TIMER	BLCA	0.3499961	0.0213897
TIMER	Time	T.cell.CD8._TIMER	KIRC	2.48E-01	0.0915597
XCELL	Diversity	T.cell.CD8._XCELL	SARC	-5.57E-01	0.07940192
XCELL	Fitness	T.cell.CD8._XCELL	LUSC	-1.99E-01	0.07693762
XCELL	Diversity	T.cell.CD8..central.memory_XCELL	SARC	-5.89E-01	0.07940192
XCELL	Fitness	T.cell.CD8..central.memory_XCELL	LUSC	-2.68E-01	0.00931648
XCELL	Diversity	T.cell.CD8..naive_XCELL	BRCA	-3.69E-01	0.0349398
CIBERSORT	Time	T.cell.follicular.helper_CIBERSORT	UCEC	0.3796764	0.00073803
CIBERSORT	Time	T.cell.follicular.helper_CIBERSORT.ABS	UCEC	0.3048422	0.02433438
XCELL	Diversity	T.cell.gamma.delta_XCELL	BRCA	-3.54E-01	0.0349398
CIBERSORT	Diversity	T.cell.regulatory..Tregs._CIBERSORT.ABS	SARC	-0.618531	0.08517683

Reference

1. Caravagna, G., Sanguinetti, G., Graham, T. A. & Sottoriva, A. The MOBSTER R package for tumour subclonal deconvolution from bulk DNA whole-genome sequencing data. *BMC Bioinformatics* **21**, 531 (2020).
2. Heide, T. *TEMULATOR: Creation of Synthetic Tumour Sequencing Data*. (2022).
3. Grossman, R. L. *et al.* Toward a Shared Vision for Cancer Genomic Data. *N. Engl. J. Med.* **375**, 1109–1112 (2016).
4. Chandrashekar, P. *et al.* Somatic selection distinguishes oncogenes and tumor suppressor genes. *Bioinformatics* **36**, 1712–1717 (2020).
5. Sondka, Z. *et al.* The COSMIC Cancer Gene Census: describing genetic dysfunction across all human cancers. *Nat. Rev. Cancer* **18**, 696–705 (2018).
6. Tingley, D., Yamamoto, T., Hirose, K., Keele, L. & Imai, K. mediation: R Package for Causal Mediation Analysis. *J. Stat. Softw.* **59**, 1–38 (2014).
7. Martincorena, I. *et al.* Universal Patterns of Selection in Cancer and Somatic Tissues. *Cell* **171**, 1029-1041.e21 (2017).
8. Cannataro, V. L., Gaffney, S. G. & Townsend, J. P. Effect Sizes of Somatic Mutations in Cancer. *JNCI J. Natl. Cancer Inst.* **110**, 1171–1177 (2018).
9. A practical guide for mutational signature analysis in hematological malignancies | Nature Communications. https://www.nature.com/articles/s41467-019-11037-8.
10. Wang, S. *et al.* Copy number signature analysis tool and its application in prostate cancer reveals distinct mutational processes and clinical outcomes. *PLOS Genet.* **17**, e1009557 (2021).

11. Wang, S., Tao, Z., Wu, T. & Liu, X.-S. Sigflow: an automated and comprehensive pipeline for cancer genome mutational signature analysis. *Bioinformatics* **37**, 1590–1592 (2021).

12. Blokzijl, F., Janssen, R., van Boxtel, R. & Cuppen, E. MutationalPatterns: comprehensive genome-wide analysis of mutational processes. *Genome Med.* **10**, 33 (2018).

13. Xu, S. *et al.* Using clusterProfiler to characterize multiomics data. *Nat. Protoc.* **19**, 3292–3320 (2024).

14. Kanehisa, M. & Goto, S. KEGG: Kyoto Encyclopedia of Genes and Genomes. *Nucleic Acids Res.* **28**, 27–30 (2000).

15. Ahmadinejad, N. *et al.* Accurate Identification of Subclones in Tumor Genomes. *Mol. Biol. Evol.* **39**, msac136 (2022).

16. Weber, S., Li, Y., Iii, J. W. S., Kakizume, T. & Schmidli, H. Applying Meta-Analytic-Predictive Priors with the R Bayesian Evidence Synthesis Tools. *J. Stat. Softw.* **100**, 1–32 (2021).

17. Bauer, D. F. Constructing Confidence Sets Using Rank Statistics. *J. Am. Stat. Assoc.* **67**, 687–690 (1972).

18. Hollander, M., Wolfe, D. A. & Chicken, E. *Nonparametric Statistical Methods*. (John Wiley & Sons, 2013).

19. Cook, J. Numerical computation of stochastic inequality probabilities. *UT MD Anderson Cancer Cent. Dep. Biostat. Work. Pap. Ser.* (2003).

20. Dentro, S. C. *et al.* Characterizing genetic intra-tumor heterogeneity across 2,658 human cancer genomes. *Cell* **184**, 2239-2254.e39 (2021).

21. Wagle, N. *et al.* The Metastatic Breast Cancer (MBC) project: Accelerating translational research through direct patient engagement. *J. Clin. Oncol.* **35**, 1076–1076 (2017).

22. Wintersinger, J. A. *et al.* Reconstructing Complex Cancer Evolutionary Histories from Multiple Bulk DNA Samples Using Pairtree. *Blood Cancer Discov.* **3**, 208–219 (2022).

23. Dymerska, D. & Marusiak, A. A. Drivers of cancer metastasis – Arise early and remain present. *Biochim. Biophys. Acta BBA - Rev. Cancer* **1879**, 189060 (2024).

24. Hu, Y., Yu, X., Xu, G. & Liu, S. Metastasis: an early event in cancer progression. *J. Cancer Res. Clin. Oncol.* **143**, 745–757 (2016).

25. Friberg, S. & Nyström, A. Cancer Metastases: Early Dissemination and Late Recurrences. *Cancer Growth Metastasis* **8**, 43–49 (2015).
